# Optical visualisation of thermogenesis in stimulated single-cell brown adipocytes

**DOI:** 10.1038/s41598-017-00291-9

**Published:** 2017-05-03

**Authors:** Rókus Kriszt, Satoshi Arai, Hideki Itoh, Michelle H. Lee, Anna G. Goralczyk, Xiu Min Ang, Aaron M. Cypess, Andrew P. White, Farnaz Shamsi, Ruidan Xue, Jung Yeol Lee, Sung-Chan Lee, Yanyan Hou, Tetsuya Kitaguchi, Thankiah Sudhaharan, Shin’ichi Ishiwata, E. Birgitte Lane, Young-Tae Chang, Yu-Hua Tseng, Madoka Suzuki, Michael Raghunath

**Affiliations:** 1 Department of Biomedical Engineering, National University of Singapore, 117583 Singapore, Singapore; 2 NUS Tissue Engineering Program, Life Science Institute, National University of Singapore, 117510 Singapore, Singapore; 3 NUS Graduate School for Integrative Sciences and Engineering (NGS), National University of Singapore, 117456 Singapore, Singapore; 4 WASEDA Bioscience Research Institute in Singapore (WABIOS), 138667 Singapore, Singapore; 5 Organization for University Research Initiatives, Waseda University, Tokyo, 162-0041 Japan; 6 Department of Pure and Applied Physics, Graduate School of Advanced Science and Engineering, Waseda University, Tokyo, 169-8555 Japan; 7 Epithelial Biology Laboratory, Institute of Medical Biology (IMB), Agency for Science, Technology and Research (A*STAR), 138648 Singapore, Singapore; 8 Section on Integrative Physiology and Metabolism, Joslin Diabetes Center, Harvard Medical School, Boston, MA 02215 USA; 9 Diabetes, Endocrinology, and Obesity Branch, National Institute of Diabetes and Digestive and Kidney Diseases, National Institutes of Health, Bethesda, MA 20892 USA; 10 Department of Orthopedic Surgery, Beth Israel Deaconess Medical Center, Harvard Medical School, Boston, MA 02215 USA; 11 Department of Chemistry & MedChem Program of Life Sciences Institute, National University of Singapore, 117543 Singapore, Singapore; 12 Laboratory of Bioimaging Probe Development, Singapore Bioimaging Consortium (SBIC), Agency for Science, Technology and Research (A*STAR), 138667 Singapore, Singapore; 13 Institute of Medical Biology, Agency for Science, Technology and Research (A*STAR), 138648 Singapore, Singapore; 14 Department of Physics, Faculty of Science and Engineering, Waseda University, Tokyo, 169-8555 Japan; 15 PRESTO, Japan Science and Technology Agency, Saitama, 332-0012 Japan; 16 Department of Biochemistry, Yong Loo Ling School of Medicine, National University of Singapore, 117597 Singapore, Singapore; 17 Aptabio Therapeutics Inc., Yongin City, 446-908 Korea; 18 Institute for Chemistry and Biotechnology (ICBT), Zurich University of Applied Sciences, Wädenswil, CH - 8820 Switzerland

## Abstract

The identification of brown adipose deposits in adults has led to significant interest in targeting this metabolically active tissue for treatment of obesity and diabetes. Improved methods for the direct measurement of heat production as the signature function of brown adipocytes (BAs), particularly at the single cell level, would be of substantial benefit to these ongoing efforts. Here, we report the first application of a small molecule-type thermosensitive fluorescent dye, ERthermAC, to monitor thermogenesis in BAs derived from murine brown fat precursors and in human brown fat cells differentiated from human neck brown preadipocytes. ERthermAC accumulated in the endoplasmic reticulum of BAs and displayed a marked change in fluorescence intensity in response to adrenergic stimulation of cells, which corresponded to temperature change. ERthermAC fluorescence intensity profiles were congruent with mitochondrial depolarisation events visualised by the JC-1 probe. Moreover, the averaged fluorescence intensity changes across a population of cells correlated well with dynamic changes such as thermal power, oxygen consumption, and extracellular acidification rates. These findings suggest ERthermAC as a promising new tool for studying thermogenic function in brown adipocytes of both murine and human origins.

## Introduction

Warm-blooded animals (or endotherms) have developed a number of metabolic processes for thermogenesis to maintain an optimal body temperature, even under extreme cold conditions^1^. Skeletal muscle tissue generates heat via shivering (involuntary tremor of the muscle)^2^ or uncoupling of sarco/endoplasmic reticulum Ca^2+^-ATPase (SERCA)-mediated ATP hydrolysis from Ca^2+^ transport^3^. These processes can generate substantial amounts of heat; however, they are insufficient to maintain body temperature under specific conditions, such as in newborns or during sustained cold exposure. Thus, a parallel mechanism has evolved in brown adipose tissue (BAT) to protect hibernating and newborn animals. Non-shivering thermogenesis in brown adipocytes (BAs) occurs via activation of uncoupling protein 1 (UCP1), which is located in the inner mitochondrial membrane and uncouples oxidative phosphorylation (and consequently ATP production) from respiration, resulting in increased heat production^4, 5^. The recent finding that BAT is not only present in neonates and small infants but also in adults^6–11^, along with the enhanced capacity of BAT for substrate combustion during non-shivering thermogenesis^12^, has generated substantial interest in BAT as a potential target for treatment of obesity and diabetes.

A key step for the evaluation of pharmaceutical and nutraceutical modulation of BA activity is the ability to monitor changes in BAT function. While the direct measurement of heat production would seem the most appropriate physiological read-out, developing appropriate methodologies has proven surprisingly challenging. Instead, indirect methods, such as mitochondrial depolarisation and oxygen consumption, are typically employed to quantitate heat production-related phenomena in differentiated adipocytes^13^. A number of techniques for the measurement of BAT thermal power have been introduced recently, including infrared thermography of activated human BA cultures^14–16^, the use of a bimaterial microcantilever, which is able to monitor small temperature variations (0.2 K) in the vicinity of norepinephrine-stimulated primary mouse BAs^17^, and microcalorimetry^18–20^. However, these techniques are not yet widely applied in metabolic research, most likely as a result of the high cell requirements and the inability to measure individual isolated primary cells. A new multi-channel isothermal microcalorimeter (calScreener), capable of monitoring heat production in growing bacteria, tumour micro-tissues, parasitic worms^21^ and soil-transmitted helminths^22^ in a convenient well-plate format has been introduced recently; however, this has not yet been tested with BAs.

As a means of measuring temperature changes at a single-cell scale, fluorescent thermosensors, capable of monitoring intracellular temperature changes in various cell types^23–35^, have also been described. To date, only two thermosensors, a genetically encoded fluorescent protein^31^ and a cytoplasmic nanogel^25^ have been used for measuring BA thermogenesis. However, both methods require laborious procedures (adenovirus transduction and microinjection, respectively) for probe delivery into the cell. As such, a more user-friendly small molecule-type dye^33–35^ would offer a practical advantage.

Here, we present a novel BODIPY-based thermosensitive dye, ERthermAC, with promising biocompatibility features (small size, excellent cellular uptake, low cytotoxicity, and robust photostability) that is capable of imaging heat production in individual isoproterenol (ISO)-stimulated mouse BAs and in forskolin-stimulated human BAs by confocal microscopy. Data obtained using this dye is comparable with that obtained by conventional methods measuring mitochondrial depolarisation, oxygen consumption, and extracellular acidification rates, and also with thermal power measurements obtained using the calScreener. We believe our findings will facilitate research on the mechanisms regulating brown fat thermogenesis as well as providing new *in vitro* tools for testing potential modulators of thermogenesis in this unique tissue.

## Results

### Characterisation of ERthermAC

A diversity-oriented fluorescent library (DOFL) was previously generated through combinatorial synthesis and by the modification of side chains of different fluorescent dye backbones^36^. Screening of this library for temperature sensitivity led to the recent discovery of ER thermo yellow^33^. To improve the photostability of the original dye, we removed the alpha-chlorine from ER thermo yellow forming the acetyl-derivative designated ERthermAC. For synthesis (Fig. S1A), absorption and emission spectra (Fig. S1B), NMR spectra (Fig. S2), HPLC chromatogram and mass spectrum (Fig. S3) of ERthermAC, see Supplemental Information. ERthermAC was found to be remarkably photostable, showing only negligible bleaching under harsh conditions (continuous irradiation with high laser power at 543 nm) in contrast to ER thermo yellow (Fig. S4).

**Figure 1:**
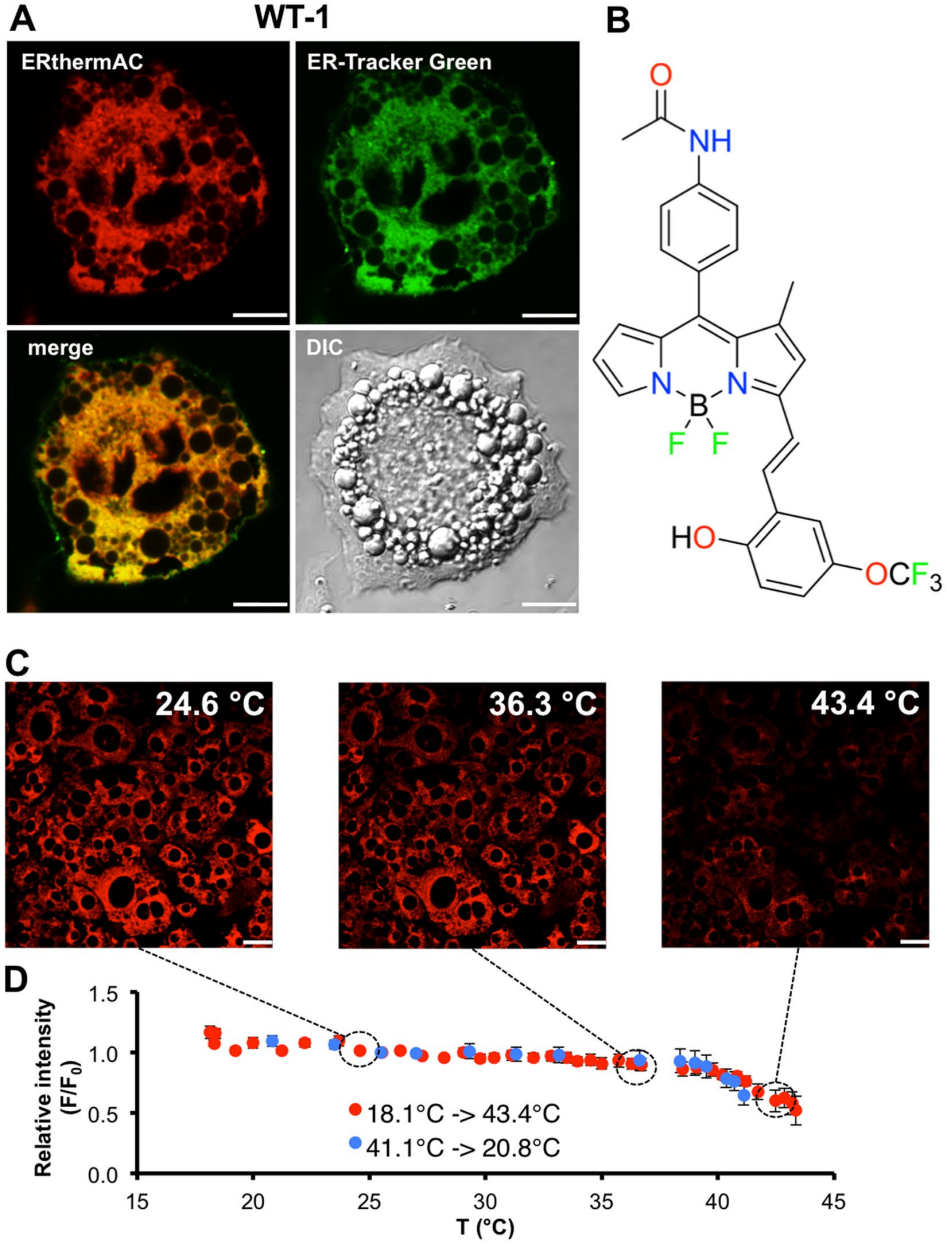
ERthermAC targets the endoplasmic reticulum in adipocytes, and its intensity is inversely proportional to temperature. (**A**) ERthermAC (red) co-localises with ER-Tracker Green (green) in WT-1 cells, as evident from the yellow mix colour after superimposition of signals. Scale bar: 10 μm. (**B**) Chemical structure of ERthermAC. (**C**) Differentiated WT-1 cells were stained with ERthermAC, fixed with 4% formaldehyde, and imaged at different temperatures ranging between 18 °C and 43 °C. At higher temperatures, cells display lower ERthermAC fluorescence intensity. Scale bar: 20 μm. (**D**) Calibration curve shows a reversible non-linear relationship between temperature and fluorescence intensity of ERthermAC in fixed WT-1 cells between 18 °C and 43 °C. Temperature sensitivities determined by the linear fit were −1.07%/°C (R^2^ = 0.76, n = 15 cells) between 18.1 °C and 35.0 °C, and −4.76%/°C (R^2^ = 0.83, n = 15 cells) between 37 °C and 43 °C.

To demonstrate that this chemical modification did not change organelle specificity, we co-stained cells with the commercially available endoplasmic reticulum dye ER-Tracker Green. Indeed, strong co-localisation of both dyes was observed in murine brown adipocytes derived from *in vitro* differentiation of the established WT-1 brown preadipocytes^37^ (Fig. 1A), confirming ER-specificity of ERthermAC (chemical structure shown in Fig. 1B) in adipocytes.

The relationship between temperature and fluorescence intensity of ERthermAC staining was assessed in formaldehyde-fixed WT-1 cells under temperature-controlled conditions between 18 °C and 43 °C (Fig. 1C and D). A linear reduction in fluorescence intensity was observed between 18.1 °C and 35.0 °C (−1.07%/°C). Fluorescence was more rapidly decreased between 35.7 °C and 42.8 °C, but this reduction also showed an approximately linear relationship (−4.76%/°C, which is comparable to of ER thermo yellow)^33^.

### Characterisation of WT-1 adipocytes

To confirm the suitability of WT-1^37^ cells as a model of BAT, we first assessed their adipogenic differentiation efficiency. Lipid production and storage was visualised using the neutral lipid-specific dye BODIPY 493/503, revealing dense intracellular lipid droplet deposition in WT-1 cells following differentiation protocols (Fig. S5A).

Adipogenesis was further assessed via quantitative real-time PCR measurement of leptin, fatty acid binding protein 4 (*fabp4*), and peroxisome proliferator-activated receptor-γ 2 (*pparg2*) mRNA. Significant increases in the expression of these pan-adipocyte markers were observed in induced WT-1 cells compared with the uninduced control group (Fig. S5B). To verify differences in the thermogenic potential of BAT cell model, we then examined the expression of brown fat-specific genes (Fig. S5C). UCP1 mRNA expression was increased ~745-fold in differentiated WT-1 brown adipocytes compared with undifferentiated cells. Gene expression levels of peroxisome proliferator-activated receptor-γ coactivator 1-α (*pgc1a*) and cell death-inducing DFFA-like effector A (*cidea*), additional markers of brown fat, were also elevated in induced WT-1 cells (~12-fold and ~3760-fold, respectively). These data suggest that WT-1 cells represent suitable *in vitro* models of BAT.

Mitochondrial respiration has been shown to correlate closely with heat production^38^. Indeed, UCP1 activation results in collapse of the electrochemical proton gradient, which in turn blocks oxidative phosphorylation. The cell attempts to restore this gradient by accelerating the electron transport chain, a process requiring increased oxygen uptake^39^. Increased mitochondrial respiration and glycolytic activity upon adrenergic stimulation were confirmed in WT-1 cells using the Seahorse Extracellular Flux Analyzer. A significant increase in oxygen consumption (OCR) (Fig. S6A) and extracellular acidification (ECAR) rates (Fig. S6B) were observed in ISO-stimulated WT-1 cells immediately after ISO addition.

Consistent with these changes in OCR and ECAR, we observed a remarkable heat-producing response in WT-1 cells upon ISO stimulation using the calScreener isothermal microcalorimeter. At 75 min post-stimulation, the average thermal power of ISO- and vehicle-stimulated WT-1 cell cultures was 22.3 μW and 15.2 μW, respectively. Over the subsequent five hours, the heat flow of both groups gradually decreased to 10.7 μW (ISO) and 9.0 μW (vehicle) (Fig. S6C). Over this period, ISO-stimulated WT-1 cells produced on average 36.5% more heat (271.7 mJ/well) compared with the vehicle group (199.1 mJ/well) (Fig. S6D).

### Fluorescence intensity of ERthermAC drastically decreases upon isoproterenol stimulation in WT-1 cells

**Figure 2:**
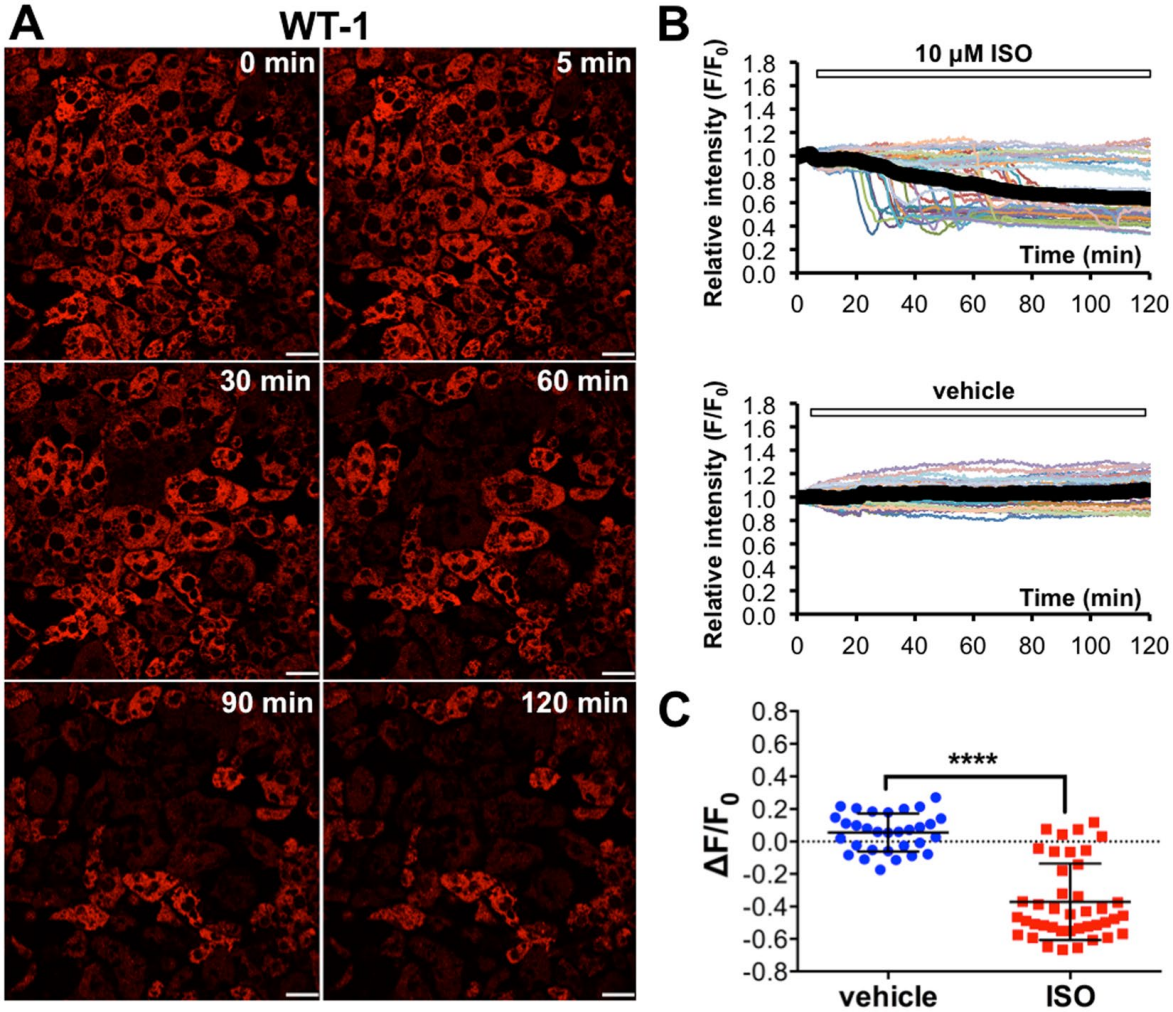
ERthermAC shows robust temperature changes after isoproterenol stimulation in WT-1 cells. (**A**) Individual ISO-stimulated WT-1 cells exhibit decreased ERthermAC fluorescence intensity, suggesting a robust increase in temperature. For time-lapse videos, see Supplementary Video 1. Scale bar: 20 ﻿µm.(**B**) Quantitative analysis of ERthermAC fluorescence intensity after ISO and vehicle stimulation: individual WT-1 cells exhibit a rapid decline in relative intensity after ISO stimulation. The reductions in fluorescence intensity occur at different time points in individual cells. In contrast, vehicle stimulation does not affect ERthermAC intensity. Thick black curves correspond to the mean relative intensities in each group. (**C**) Scatter plot of ERthermAC relative intensity change shows a significant difference in WT-1 cells. Bars show mean ± SD. WT-1 vehicle: n = 31 cells from 2 cultures; WT-1 ISO: n = 41 cells from 2 cultures.

**Figure 3:**
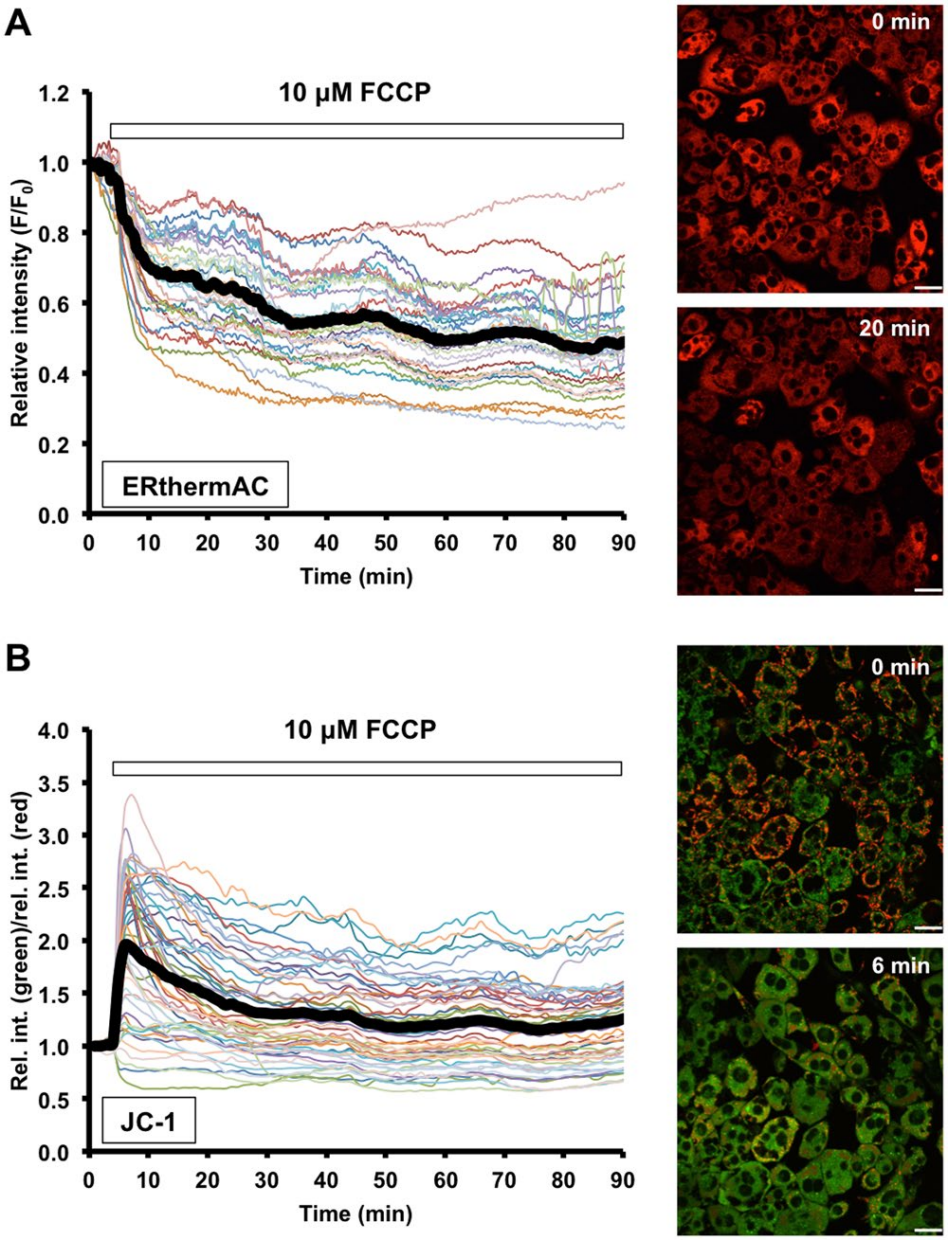
FCCP stimulation resulted in immediate effects in WT-1 cells. (**A**) ERthermAC intensity drastically decreases upon FCCP stimulation in all cells, without any lag phase, indicating increased intracellular temperature. The thick black curve corresponds to the mean relative intensity; n = 35 cells from 2 cultures. Scale bar: 20 μm. (**B**) JC-1 staining shows immediate mitochondrial depolarisation after FCCP treatment. The thick black curve corresponds to the mean relative intensity ratio of green (JC-1 monomers) and red (JC-1 aggregates) signals; n = 50 cells from 2 cultures. Scale bar: 20 μm.

Increasing temperature due to uncoupling respiration opens up competing, non-radiative relaxation pathways by altering the structural dynamics of the ERthermAC molecule that, in turn, decreases its fluorescence intensity. Upon ISO simulation, the majority of WT-1 cells displayed a rapid reduction in ERthermAC intensity (Fig. 2A and Supplementary Video 1). Stimulation with the solvent vehicle did not alter fluorescence intensity (Figs 2B and S7). The sudden decline in fluorescence intensity occurred after a lag period and at different time points in individual cells (ranging from 20 min onwards) where it reached a minimum level within 10 minutes (Fig. S8 and Supplementary Video 2). Approximately 25% of cells (11 out of 41 cells) were unaffected, suggesting that these non-responders had not acquired thermogenic capacity upon ISO simulation (Fig. 2C). In contrast, incubation with the uncoupling ionophore FCCP led to a simultaneous reduction in intensity across all cells (n = 35 cells from 2 cultures) without an apparent lag period (Figs 3A and S9). The inverse relationship between fluorescence intensity of ERthermAC and temperature indicates heat production in these cells upon adrenergic stimulation. For statistical analysis, we calculated the change in relative intensity (ΔF/F_0_) for each cell as:

$${{\rm{\Delta }}F/F}_{{\rm{0}}}={mean}\,{relative}\,{intensity}\,({last}\,{\rm{5}}\,{\min })-{mean}\,{relative}\,{intensity}\,({initial}\,{\rm{5}}\,{\min })$$

ΔF/F_0_ values of vehicle and ISO (or FCCP) stimulation were then compared by Student’s t-test with Welch’s correction. A significant reduction in fluorescent activity was observed in ISO and FCCP-stimulated WT-1 cells compared with vehicle controls (both p < 0.0001), and the average reduction in relative intensity was comparable under both stimulatory conditions (ISO: −0.427 ± 0.042; FCCP: −0.520 ± 0.029 compared with the respective control groups). We also observed a morphological change in WT-1 cells (apparent thinning of cells), which occurred in parallel with the reduction in ERthermAC intensity (Fig. S8 and Supplementary Video 2).

### Isoproterenol stimulation results in mitochondrial depolarisation in WT-1

BAT thermogenesis requires mitochondrial depolarisation; therefore, as a measure of mitochondrial depolarisation, we determined the effect of isoproterenol stimulation on the fluorescent activity of JC-1, a highly sensitive metachromatic dye-based probe for the assessment of mitochondrial membrane potential^40^. When the mitochondrion is polarised, JC-1 forms aggregates, which are retained in the mitochondrial matrix and emit light in the orange/red region of the spectrum (at a maximum absorbance of 590 nm). In depolarised mitochondria, JC-1 monomers leak into the cytosol and display green fluorescence (at a maximum absorbance of 527 nm).

**Figure 4:**
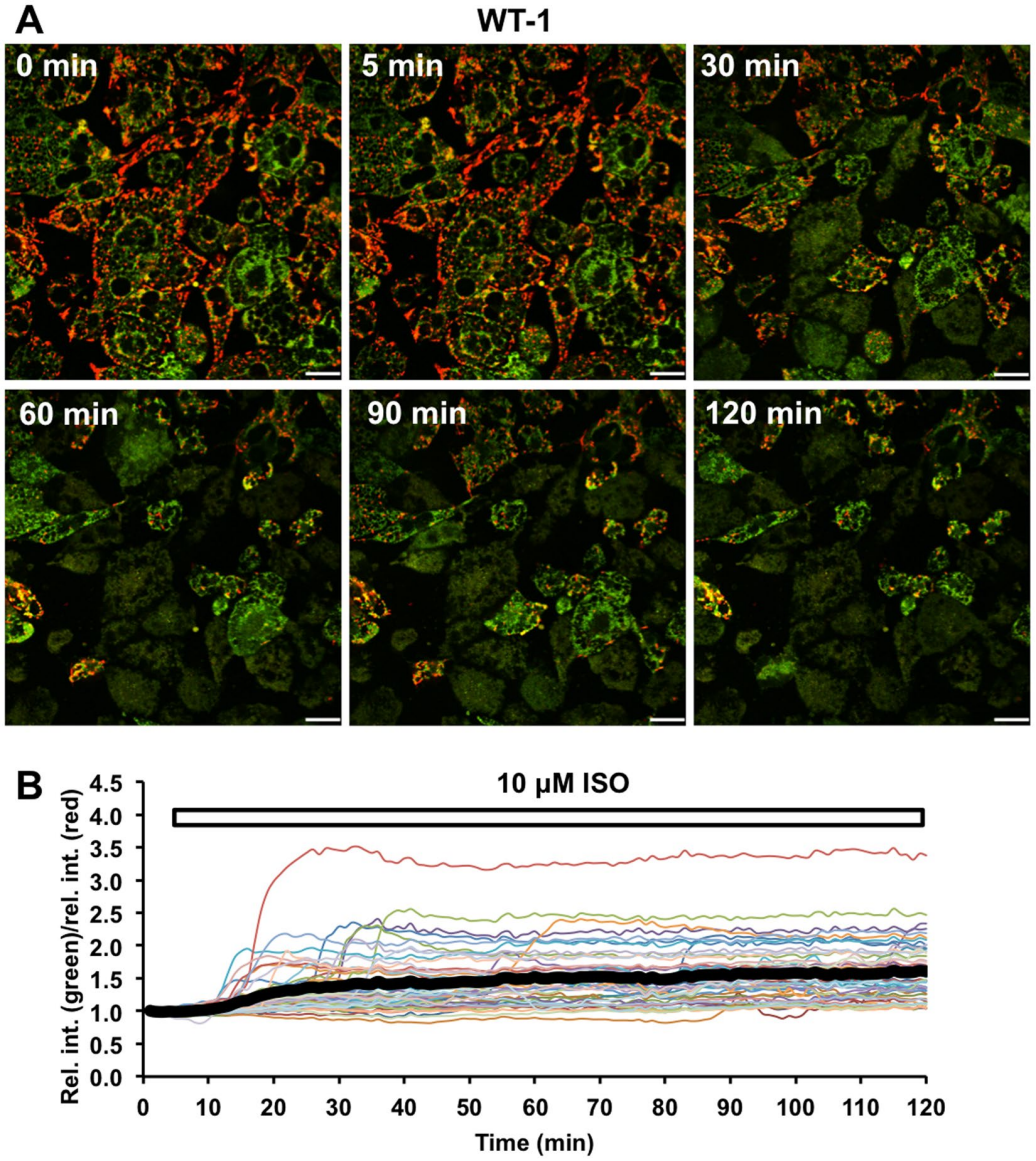
Isoproterenol stimulation induces mitochondrial depolarisation in WT-1 cells. (**A**) In WT-1 cells, red signal (JC-1 aggregates) corresponding to polarised mitochondria disappears and the intensity of green signal (JC-1 monomers) increases after ISO stimulation, indicating depolarisation. For time-lapse video, see Supplementary Video 3. Scale bar: 20 ﻿μm.(**B**) The relative intensity ratio of green and red signals is rapidly altered following ISO stimulation in WT-1 cells, indicating mitochondrial depolarisation. These sudden changes occur at different time points in individual cells. The thick black curve represents the average relative intensity ratio of green to red signals in all imaged cells, including responding and non-responding cells; n = 47 cells from 2 dishes.

FCCP stimulation induced a rapid JC-1 colour change simultaneously in all WT-1 cells with no lag period (Fig. 3B), consistent with the effects seen with ERthermAC. Moreover, in accordance with the changes observed in ERthermAC intensity in WT-1 cells following ISO stimulation, we observed a rapid red-to-green transition of JC-1 after an initial lag phase (Fig. 4, Supplementary Video 3). As before, this change occurred at different time points in individual cells (Fig. 4B). We also observed the previously noted morphological change in ISO-stimulated cells, indicating that this was not a specific effect of ERthermAC (Fig. S10 and Supplementary Video 4). Thus, the depolarisation kinetics determined using JC-1 in ISO and FCCP-stimulated WT-1 cells were comparable to the changes in ERthermAC intensity described above.

### Characterisation of human adipocytes

A new immortalized brown preadipocyte cell line was developed using methods described in Xue *et al.* ^41^. After adipogenic induction and differentiation, human brown adipocytes (hBAs) showed dense lipid droplet accumulation (Fig. S11A). Moreover, brown preadipocytes exposed to induction cocktail expressed significantly higher levels of mature adipocyte markers, such as fatty acid synthase (*FAS*), *FABP4* and *PPARG2*, compared to undifferentiated precursor cells (Fig. S11B). *UCP1*-expression was significantly higher (59,055-fold) in mature hBAs than in undifferentiated preadipocytes, suggesting high thermogenic capacity. Furthermore, mRNA levels of type II iodothyronine deiodinase (﻿*DIO2*) and *﻿PGC1A﻿* were also significantly elevated (45.8 and 8.7-fold, respectively) in hBAs compared to the undifferentiated control group (Fig. S11C).

In accordance with results obtained by WT-1 cells, forskolin stimulation enhanced respiration in mature hBAs. OCR increased to 240% of basal level and it did not decrease throughout the measurement (Fig. S12A). Moreover, forskolin stimulation resulted in increased extracellular acidification upon stimulation (Fig. S12B).

### Intensity drop of ERthermAC suggests heat production in human brown adipocytes

**Figure 5:**
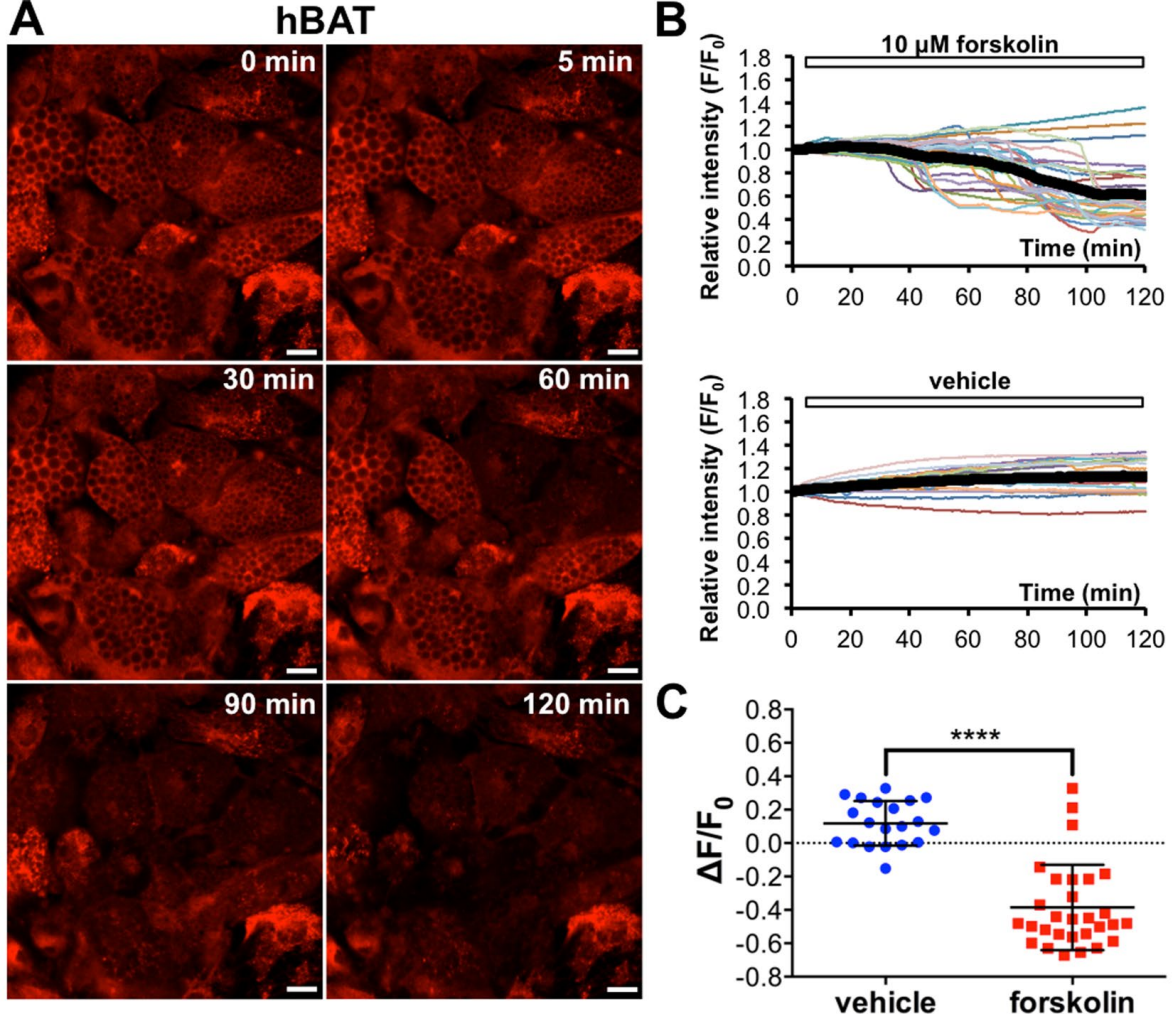
Intensity drop of ERthermAC suggests heat production in human brown adipocytes after forskolin stimulation. (**A**) Individual forskolin-stimulated human brown adipocytes (BAs) exhibit decreased ERthermAC fluorescence intensity, suggesting a robust increase in temperature. Scale bar: 20 μm. For time-lapse videos, see Supplementary Video 5. (**B**) Quantitative analysis of ERthermAC fluorescence intensity in human BAs after forskolin and vehicle stimulation: individual cells exhibit a rapid decline in relative intensity after forskolin stimulation. The reductions in fluorescence intensity occur at different time points in individual cells. In contrast, vehicle stimulation does not affect ERthermAC intensity. Thick black curves correspond to the mean relative intensity in each group. (**C**) Scatter plot of ERthermAC relative intensity change shows a significant difference between forskolin and vehicle stimulated brown adipocytes. Bars show mean ± SD. hBAT vehicle: n = 20 cells from 3 cultures; hBAT forskolin: n = 29 cells from 3 cultures.

The majority of stimulated hBAs displayed a rapid reduction in ERthermAC intensity suggesting heat production (Fig. 5A, Supplementary Video 5). Similarly to WT-1 cells, the sudden decline in fluorescence intensity occurred after a lag period and at different time points in individual hBAs. In contrast, vehicle stimulation did not cause significant alterations in ERthermAC intensity (Fig. 5B). After forskolin stimulation, two different groups of cells could be identified, corresponding to responding (89.7%) and non-responding cells (10.3%), suggesting that the differentiated human brown adipocytes represent a heterogeneous population of cells. The average amplitude of intensity drop was −0.504 ± 0.056 in stimulated hBAs, which is comparable to that of WT-1 cells. In contrast, forskolin stimulation did not result in ERthermAC intensity drop in undifferentiated stromal vascular fraction (SVF) cells from brown fat tissue (Fig. S13).

### JC-1 colour change suggests mitochondrial depolarisation in human brown adipocytes

**Figure 6:**
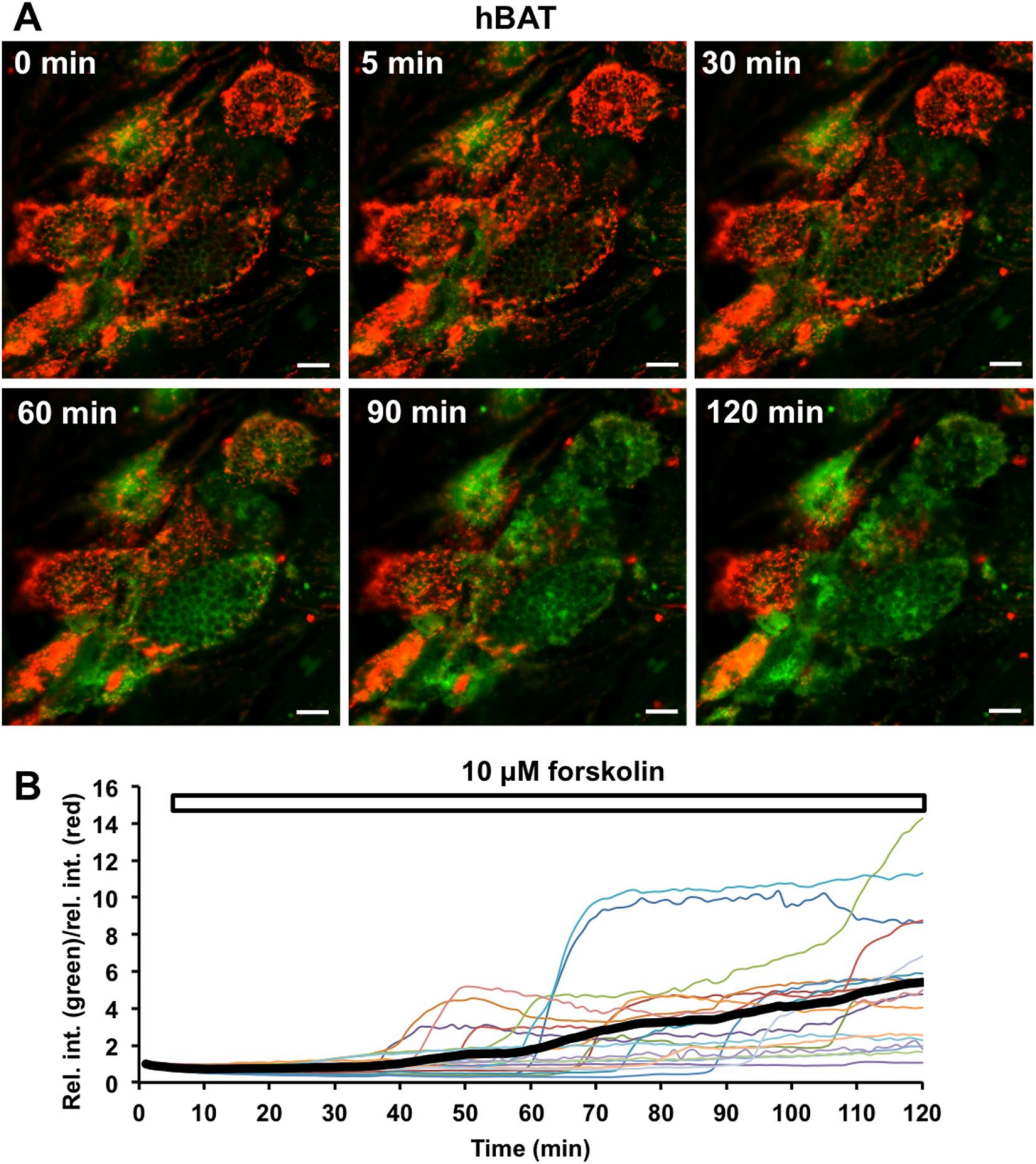
Forskolin stimulation induces mitochondrial depolarisation in human brown adipocytes. (**A**) In human brown adipocytes (BAs), red signal (JC-1 aggregates) corresponding to polarised mitochondria disappears and the intensity of green signal (JC-1 monomers) increases after forskolin stimulation, indicating depolarisation. For time-lapse video, see Supplementary Video 6. Scale bar: 20 ﻿μm. (**B**) The relative intensity ratio of green and red signals is rapidly altered following forskolin stimulation in human BAs, indicating mitochondrial depolarisation. These sudden changes occur at different time points in individual cells. The thick black curve represents the average relative intensity ratio of green to red signals in all imaged cells, including responding and non-responding cells.

Similarly to WT-1 cells, we observed a rapid red-to-green transition of JC-1 in hBAs (Fig. 6A, Supplementary Video 6), which change occurred at different time points in individual cells (Fig. 6B). These findings suggest that forskolin stimulation results in mitochondrial depolarisation in human brown adipocytes.

### Changes in endoplasmic reticulum morphology, Ca^2+^ concentration and pH do not underlie the changes in ERthermAC fluorescence intensity

We then considered whether ERthermAC intensity might be affected by other major and dynamic cellular factors, such as morphology, Ca^2+^ concentration, and pH. Distribution and intensity of ER-Tracker Green was unaltered following ISO stimulation in WT-1 cells (Fig. S14), indicating that changes in cell morphology were unlikely to contribute to the observed decrease in ERthermAC fluorescence intensity. The effect of Ca^2+^ on ERthermAC intensity was assessed by incubation in HEPES buffer containing a range of physiological Ca^2+^ concentrations (10–1000 μM)^42^ by spectrophotometry. No change in fluorescence intensity was observed across this range (Fig. S15).

To estimate pH fluctuations in the ER of ISO-stimulated WT-1 cells, we used a super ecliptic pHluorin^43^ tagged with an ER-targeting KDEL signal peptide (for ER-pHluorin probe generation, see Supplemental Information). We observed a biphasic pH profile in response to ISO stimulation (Fig. S16). After an initial gradual decrease from basal levels (pH 7.3 ± 0.1) to pH 7.0 ± 0.1, most likely a result of fatty acid release during lipolysis^44^, the intra-endoplasmic pH was rapidly and substantially increased to pH 8.1 ± 0.3. Notably, this increase coincided with both the previously noted morphological changes as well as the respective changes in intensity and colour of ERthermAC and JC-1. However, determination of the relationship between pH and ERthermAC fluorescence using the same method as for ER-pHluorin sensor calibration^45^ revealed that fluorescence intensity was only reduced by approximately 8.5% between pH 7.0 and 8.1 (Fig. S16E; for detailed calibration protocol, see Supplemental Information). For comparison, the average ERthermAC intensity reduction in WT-1 cells was 42.7% following ISO stimulation, suggesting that alkalisation was not a major contributing factor to the signal changes observed.

## Discussion

Thermogenesis is a crucial physiological task for endothermic animals, and brown adipose tissue, in particular, has the capacity to produce high levels of heat via uncoupled respiration. Originally, BAT was assumed to be present only in human newborns and small infants to provide shiver-free thermogenesis, but has now also been shown to be present in adults^6–11^. This discovery has been the subject of substantial interest in both academic and pharmaceutical research^46^ as a result of the high level of glucose and free fatty acid consumption by activated murine BAT – it is estimated that 50 g of BAT can burn 250 kcal daily^47^. Stimulation of BAT energy expenditure via sympathetic^48^ or non-sympathetic^49, 50^ pathways is considered a potential new drug target for obesity and diabetes treatments, and there is increasing interest in the discovery of novel mechanisms and substances for this purpose (reviewed in ref. 51). However, to facilitate the development of breakthrough therapies, it is essential that techniques to measure the signature function of BAT are readily available.

**Figure 7:**
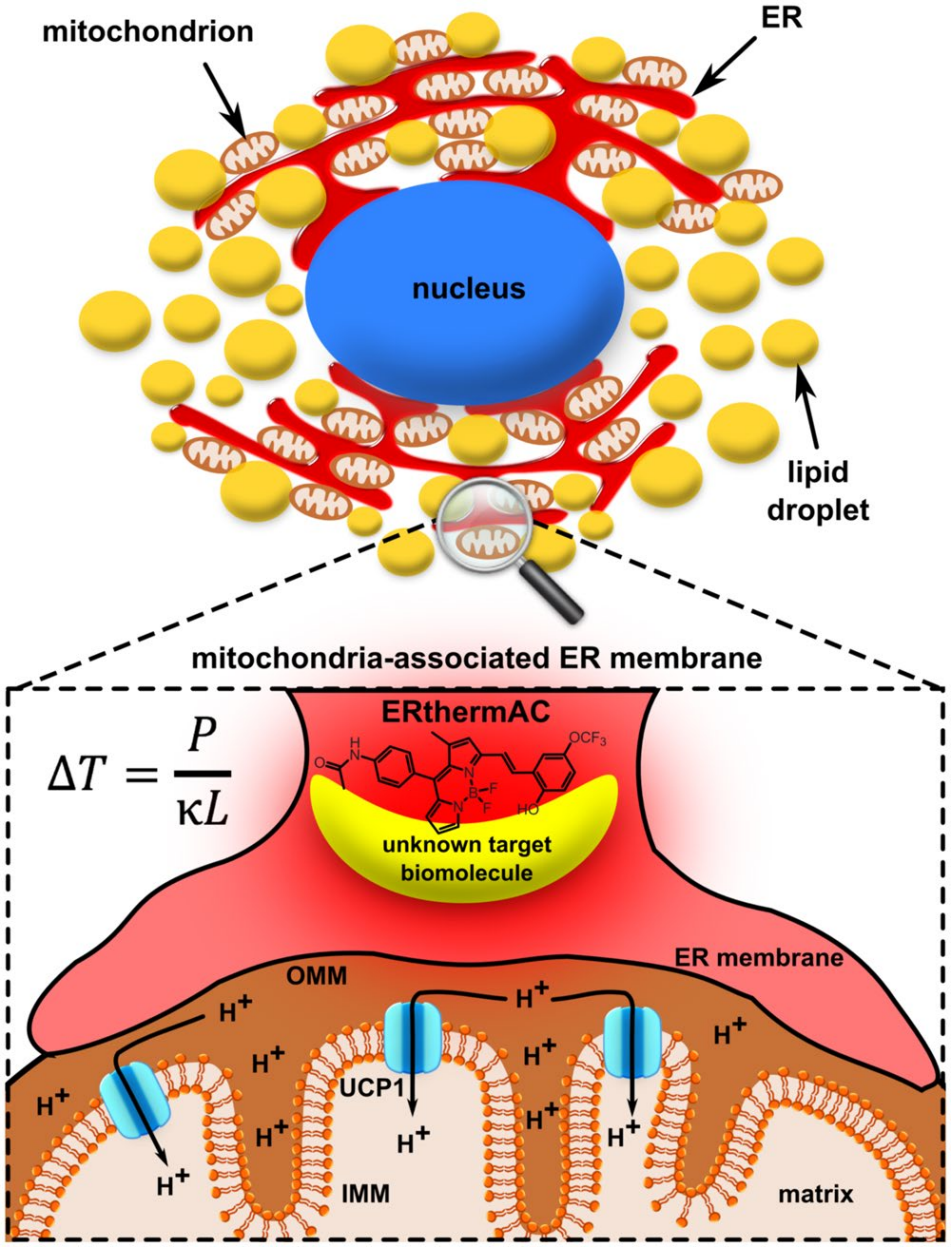
ERthermAC forms a contiguous thermometer in the immediate mitochondrial vicinity by targeting the endoplasmic reticulum. Temperature difference (*ΔT* [K]) is proportional to the power of the heat source (*P* [W]), and inversely proportional to the distance (*L* [m]) from the centre of the heat source and the thermal conductivity surrounding the heat source (κ [W m^−1^ K^−1^]). Mitochondria-associated ER membrane in adipocytes provides sufficiently close proximity of these two organelles, thus placing ERthermAC into the extensive endoplasmic network and creating an extended contiguous thermometer in the immediate mitochondrial vicinity through targeting of a yet unknown biomolecule. This process is unaffected by radical environmental changes in mitochondria. ER: endoplasmic reticulum; IMM: inner mitochondrial membrane; OMM: outer mitochondrial membrane; UCP1: uncoupling protein 1.

Intracellular thermosensors, including thermoresponsive polymers^23–26^, dye-doped polymeric nanoparticles^27–29^, fluorescent proteins^30, 31^, and small molecules^32–35^, which are capable of monitoring intracellular temperature changes at the single-cell level have been described previously. Here, we used a newly developed small molecule-type thermosensitive dye, ERthermAC, that is easily taken up by cells within 30 minutes and accumulates rapidly and specifically in the ER. Although activation of UCP1 takes place in the mitochondria, we positioned ERthermAC into the endoplasmic reticulum for several reasons. Firstly, the chemical and physical properties of mitochondria, such as membrane potential, can vary extensively under thermogenic stimulation, potentially impairing accuracy of measurements. In contrast, ER membrane potential remains minimally depolarised during Ca^2+^ release^52^. Physiological Ca^2+^ concentrations (10–1000 μM)^42^ were not found to influence ERthermAC intensity, and pH, which varies within the ER between 7.0 and 8.1 during adrenergic stimulation, had only a modest impact (−8.5%) on its fluorescence intensity. Secondly, mitochondria and ER are in close proximity in BAs, with lipid droplets occupying the majority of intracellular space. Recent evidence suggests that ER membranes are fused with the outer mitochondrial membrane in BAT^53^, which would not only provide opportunities for intensive communication between these organelles but also sufficiently close proximity for heat measurement. Thus, placing ERthermAC into the extensive endoplasmic network creates an extended contiguous thermometer in the immediate mitochondrial vicinity while reducing the potential for interference from radical environmental changes (Fig. 7).

We assessed the application of ERthermAC intensity as readout of thermogenesis using the adrenergic stimulus, isoproterenol or forskolin. ISO mimics the physiological activation of BAT via binding to beta-adrenergic receptors and the subsequent activation of adenylate cyclase^54^ and generation of second messenger cyclic adenosine monophosphate (cAMP), which in turn activates the cAMP-dependent protein kinase A (PKA). In contrast, forskolin activates adenylate cyclase directly resulting in elevated cAMP levels. PKA can phosphorylate lipolytic enzymes, such as hormone-sensitive lipase, resulting in the release of free fatty acids (FFA) from lipid droplets. In activated BAs, FFAs interact with and activate UCP1, which short-circuits the proton gradient by shunting protons away from ATP synthesis^55^. This results in a gradient discharge with heat generated by the dissipation of the proton-motive force and also indirectly by elevated respiratory chain activity in an attempt to sustain the electrochemical gradient and ATP production^46^. Adrenergic stimulation of individual BAs differentiated from immortalised mouse^37^ and human precursor cells resulted in a rapid decline in intracellular ERthermAC intensity after a lag phase of several minutes, indicating a steep increase of temperature within the immediate vicinity of the dye. In contrast, addition of the chemical uncoupler FCCP induced an immediate cellular response in all WT-1 cells. Classical uncoupling reagents, such as FCCP, CCCP and 2,4-dinitrophenol, are lipid-soluble weak acids that directly uncouple mitochondrial oxidative phosphorylation by diverting protons across the inner mitochondrial membrane. The parallel transport leading to ATP production is short-circuited, and the energy of proton motive force is dissipated as heat^56, 57^. Thus, we assume the lag phase observed with ISO stimulation may correspond to the time needed to complete the adrenergic signalling cascade. However, further work is required to determine the basis of the staggered thermogenic activation kinetics in individual cells.

Notably, the kinetics of mitochondrial membrane depolarisation, as visualised using JC-1, were comparable to those of ERthermAC staining intensity in both ISO- and FCCP-exposed WT-1 cells and in forskolin stimulated human brown adipocytes including the staggered activation of cells. Overlapping emission spectra of both fluorescent probes precluded co-staining, but the respective colour and intensity profiles strongly suggest that the same thermogenic events were captured. Moreover, bright-field image series taken in parallel in the same cell culture with either ERthermAC or JC-1 staining revealed a fast, shrinkage-like cellular movement in activated BA that occurred in concert with the intensity reduction of ERthermAC and the colour change of JC-1, strengthening the hypothesis that the same cellular event was captured with ERthermAC and JC-1.

To validate the detection of thermogenesis in our model, we further employed the current standard Seahorse XF Analyzer and the novel multi-channel calScreener microcalorimeter on WT-1 cells as alternative measures. The profiles of enhanced oxygen consumption (OCR) and extracellular acidification (ECAR) rates (Fig. S6) in ISO-stimulated WT-1 cells corresponded closely with the averaged intensity change of ERthermAC (Fig. 2) and colour change of JC-1 (Fig. 4). In addition, the multi-channel design of the calScreener allowed us to simultaneously monitor heat generation in 32 different samples of adherent cell monolayers. Thus, we were able to compare the metabolic responses of differentiated mouse BAs in real-time, similarly to Seahorse measurements. Compared to the vehicle group, isoproterenol significantly increased the thermal power of WT-1 cells by 47.1% 75 minutes after stimulation and this increase was sustained for at least 6 hours post-stimulation, although the difference between treatment groups decreased gradually over time. We determined the thermal power of WT-1 cells to be 0.5 nW per cell in a given monolayer at 75 min post-stimulation, assuming that the number of cells was not significantly changed between seeding and the calorimetric measurement. This is within the range of previous studies that measured large amounts of suspended cells in a batch microcalorimeter (0.82 nW per hamster BAs and 5.1 nW per rat BAs)^18, 19^ or using nanocalorimetric sensors (1.6 nW per mouse BAs)^20^. However, technical limitations with the current calScreener detection instrumentation, including the 3-step equilibrium procedure after administration of the stimulus and the manual closures of the detection vials, mean that the first 75 min after stimulation, and thus the initial peak of thermogenesis, are not captured, potentially leading to an underestimation of thermal power. This limitation could be overcome with the use of an injection system to allow instantaneous monitoring of heat generation.

The question of temperature heterogeneity and the interpretation of single cell thermometry data is the subject of on-going debate in the field of cellular thermosensing^58–61^, and a recent commentary proposed that temperature differentials measurable in cells should not exceed a μK range^58^. As such, the apparent temperature increase from 25 °C to 42 °C in WT-1 cells following isoproterenol stimulation as measured by ERthermAC staining would appear, at first sight, surprising (assuming the initial temperature in the ER is equivalent to that of the culture medium at 25 °C). However, it is important to note that this finding is specific to the ER and does not mean that the whole cell would reach this temperature. Site-specific thermometry close to the point of heating i.e. probably the mitochondria in the case of uncoupled respiration allows the monitoring of local temperature changes and would show smaller temperature differentials further away from the point of heating^60^. Indeed, Kiyonaka *et al.* identified a 5 °C temperature change when a recombinantly expressed thermosensitive protein targeted the mitochondria but smaller differences when the thermosensor was located in the cytosol^31^. Moreover, a recent study has reported a maximum change of 1.3 °C in the cytoplasm of stimulated brown adipocytes^25^. Our results obtained by microcalorimetry, applied for the first time in adherent monolayer cultures, along with previous findings in other cell systems^18–20^, strengthen the hypothesis that local intracellular temperatures within BAs can increase by several degrees. Furthermore, when we take into consideration that not every cell is stimulated (approximately 75% of WT-1 cells showed changes in ERthermAC intensity within 2 hours), an average thermal power of individual cells in the range of several hundred picowatts to nanowatts would provide additional support to explain our findings with ERthermAC.

It is also important to note that brown adipose tissue thermogenesis has a major role in hibernating animals during arousal when they are able to restore their body temperature from 4 °C, or even below this, to normal levels^62^, thus requiring a very large increase in heat generation. In UCP1-ablated mice, peak rewarming rates from induced torpor were reduced by 50% (UCP1^+/+^: 0.24 ± 0.08 °C min^−1^; UCP1^−/−^: 0.12 ± 0.04 °C min^−1^)^63^, strengthening the hypothesis that UCP1-facilitated non-shivering thermogenesis in BAT provides an extraordinarily high heat output to allow fast rewarming of the animal. A number of protective mechanisms exist in BAs to prevent cellular damage during these fast and relatively high temperature changes and ensure that hibernating animals endure little to no damage to their tissues during torpor arousal, such as antioxidants and heat shock proteins^64^. Moreover, it was recently demonstrated that membrane lipids, such as cardiolipin, stabilise UCP1, increasing its thermal stability^65^ and conferring a higher temperature tolerance for mitochondrial membrane proteins.

Although our results suggest that Ca^2+^ concentration or morphology have no significant effect on ERthermAC staining intensity, and the pH fluctuation in the ER is only responsible for a slight change, we cannot rule out the possibility that other unknown factors might contribute to the high intensity change detected. Further experiments using fluorescence lifetime imaging or the development of a ratiometric dye based on ERthermAC are needed to clarify the exact amount of temperature change. Nevertheless, we believe that ERthermAC provides an elegant and user-friendly method for the qualitative study of heat generation in BAs at a single-cell level.

In summary, we have successfully demonstrated that the novel BODIPY-based thermosensitive dye, ERthermAC, is quickly and easily taken up into the endoplasmic reticulum of adipocytes where it forms a contiguous intraorganellar thermometer for the optical visualisation of thermogenesis. Following adrenergic stimuli, heat production occurred at random timings in each cell, and the dynamics of thermogenesis was consistent with mitochondrial depolarisation observed using JC-1. Consequently, ERthermAC is a promising tool for examination of brown adipose tissue thermogenesis, which is compatible with both time-lapse studies and image-based high-content screening.

## Materials and Methods

### Cell culture and adipogenic induction of WT-1 brown preadipocytes

Immortalised brown preadipocytes (WT-1)^37^ were cultured in high glucose DMEM (HG DMEM) supplemented with GlutaMAX, 10% FBS (#41F5724, #41F1623K and 42F7254K; 10270, Gibco/Life Technologies, Carlsbad, CA, USA), and Penicillin/Streptomycin (P/S; Gibco/Life Technologies). Cells were maintained at 37 °C in a humidified atmosphere of 5% CO_2_. To prevent spontaneous differentiation, cells were maintained at subconfluent levels prior to being detached using TrypLE Express (Gibco/Life Technologies), passaged at 1:10, and cultured to generate subsequent passages. Directed differentiation was carried out with cells at passage 28 to 38. This cell line was used as a BAT model in our experiments.

For adipogenic induction, WT-1 cells were seeded at a density of 3 × 10^4^ cells/cm^2^ in basal medium (HG DMEM supplemented with GlutaMAX (Gibco/Life Technologies), 2% FBS, and P/S). Cells were pre-treated for three days with 3.3 nM BMP7 (354-BP, R&D Systems, Minneapolis, MN, USA), 20 nM insulin (91077 C, Sigma, St-Louis, MO, USA), and 1 nM T3 (T5516, Sigma). Cells were then exposed to an induction cocktail for two days, which consisted of 0.5 mM 3-isobutyl-1-methylxanthine (IBMX), 0.125 mM indomethacin, 5 μM dexamethasone, 20 nM insulin (I5879, I7378, D4902 and 91077 C, respectively, Sigma), and 1 nM T3 in basal medium, followed by a three-day maintenance phase (basal medium containing 20 nM insulin and 1 nM T3). Cells were cultured in HG DMEM without FBS and hormonal supplements for 24 h prior to experimental analysis. Non-induced control cells for qPCR analyses were maintained with basal medium alone on the same schedule.

### Generation of immortalized human brown preadipocytes

This study was carried out in accordance with the institutional guidelines of and was approved by the Human Studies Institutional Review Boards of Beth Israel Deaconess Medical Center and Joslin Diabetes Center. Details on procedures of human subject collection were described previously^41, 66^. For characterisation and confocal microscopy experiments, neck fat from one subject was studied. The subject gave written informed consent before taking part in the study.

Isolation and immortalization procedure of primary stromal vascular fraction (SVF) from human neck fat was described previously^66, 67^. Briefly, deep neck fat was collected from a female subject (age 56, BMI 30.8). SVF cells were isolated and expanded in culture and split a few times before immortalization. To immortalize, hBAT-SVF cells were infected with retroviral particles encoding the plasmid pBABE-Hygro-hTERT (Addgene Plasmid #1773, Cambridge, MA, USA). Following retrovirus infection, cells were selected with 200 ug/ml Hygromycin (hBAT) for two weeks. Once drug selection was finished, immortalized cells were allowed to grow in HG DMEM medium containing 10% FBS.

### Cell culture and adipogenic induction of human brown adipocytes

For adipogenic induction ﻿[68], cells were seeded in an initial density of 10.5 k cells/cm^2^. When reached confluence, the cells were pre-treated with 3.3 nM BMP7, 0.5 μM insulin and 2 nM T3 in high glucose DMEM,10% FBS and 1% P/S for 6 days. Then, the cells were treated with adipogenic induction cocktail for 18 days (medium was replaced every 3 days). The induction cocktail consisted of basal media (high glucose DMEM, 10% FBS and 1% P/S), 0.5 μM insulin, 0.1 μM dexamethasone, 30 μM indomethacine, 0.5 mM IBMX, 2 nM T3, 1 μM rosiglitazone, 33 μM biotin and 17 μM pantothenate. Non-induced control cells for qPCR analyses were maintained with basal medium alone on the same schedule. Cells were cultured in HG DMEM without FBS and hormonal supplements for 24 h prior to experimental analysis.

### Staining with different fluorescent probes

Cells were cultured and differentiated as described above in glass-bottom dishes. Mature cells were incubated with different fluorescent probes (alone or in combination) for 30 min at 37 °C in HG DMEM at the following concentrations: 250 nM ERthermAC, 500 nM ER-Tracker Green (E34241, Molecular Probes, Life Technologies), 5 μM JC-1 (T3168, Molecular Probes, Life Technologies). Medium was replaced with fresh HG DMEM prior to imaging.

For assessment of lipid droplet content, cell cultures were rinsed with PBS, fixed in 4% formaldehyde (28908, Pierce Biotechnology Inc., Rockford, IL, USA) for 10 min at room temperature, then co-stained for 30 min with 0.1 μg/ml BODIPY 493/503 (D3922, Molecular Probes, Life Technologies) for cytoplasmic lipid droplets, and 0.5 μg/ml of 4′,6-diamidino-2-phenylindole (DAPI) (D3571, Molecular Probes, Life Technologies) for nuclear DNA.

### Confocal imaging

Stained WT-1 cells were imaged with an Olympus FV 1000 confocal microscope with a 60× objective (PLAPON60XO, NA 1.42, oil immersion), equipped with stage incubator and CO_2_ supply. Microscopic images of ERthermAC and the red channel of JC-1 were obtained using a 543 nm laser (1.71 μW), dichroic mirrors DM405/488/543 and SDM560, and the emission band set at 555–655 nm. To capture ER-Tracker Green, BODIPY 493/503, ER-pHluorin, and the green channel of JC-1 images, a 488 nm laser (4.97 μW) and a dichroic mirror DM405/488/543 were used with the emission band set at 500–530 nm. Laser power was measured at the specimen using a laser power meter (NOVA II, 7Z01550, Ophir Optronics Solutions, Israel) and sensor (PD300, 7Z02410, Ophir Optronics Solutions).

For time-lapse imaging experiments (ERthermAC and JC-1), medium was replaced with 900 μl fresh HG DMEM (25 °C) after staining and equilibrated at 25 °C for 15 min. Imaging was initiated and 100 μl isoproterenol (ISO, final concentration: 10 μM; I6504, Sigma), carbonyl cyanide *p*-(tri-fluromethoxy)phenyl-hydrazone (FCCP, final concentration: 10 μM: XF Cell Mito Stress Test Kit, 101706–100, Seahorse Bioscience), or vehicle (HG DMEM) was added after 5 min. Cells were recorded over 120 min (ISO) or 90 min (FCCP), and images were acquired every 20 sec (ERthermAC) or 1 min (JC-1). Imaging speed was 4 μs/pixel; image size was 512 by 512. The intensity of fluorescence was analysed using ImageJ software. Regions of interest (ROI) were selected manually and pixel intensities were spatially averaged. Gain was set individually for each sample to avoid saturation of the signal. Consequently, results are interpreted as relative intensity (intensity of all ROIs divided by intensity at time 0).

Stained human brown adipocytes were imaged with a Zeiss LSM710NLO confocal microscope in point scan mode with a 40× objective (LD C-Apochromat 40×/1.1 W Korr), equipped with stage incubator and CO_2_ supply. Microscopic images of ERthermAC and the red channel of JC-1 were obtained using a 561 nm diode-pumped solid-state laser and the emission band was set at 589–667 nm. To capture BODIPY 493/503 and the green channel of JC-1 images, a 488 nm Argon laser were used with the emission band set at 499–560 nm. For Hoechst 33342 nuclear staining, a Chameleon Vision (Coherent, Santa Clara, CA, USA) laser was applied with the emission band set at 425–475 nm. Time-lapse imaging experiments were performed similarly as above described with the following modifications: cells were stimulated with forskolin (final concentration: 10 μM; F6886, Sigma) or vehicle (HG DMEM with DMSO) and they were recorded over 120 min (forskolin and vehicle) and images were acquired every 20 sec (ERthermAC) or 1 min (JC-1). Pixel dwell: 1.58 μsec; image size was 512 by 512.

### Statistical analysis

Unless otherwise stated, all results are reported as mean ± standard error (SEM). Student’s t-test with Welch’s correction was used for comparison between two groups of time-lapse confocal imaging results, and Student’s t-test was used for mouse qPCR, Seahorse (both species) and calScreener experiments. For multiple pairwise comparisons (human qPCR), one-way ANOVA statistical analysis was performed followed by Tukey Multiple Comparisons Test using Graphpad Prism software (GraphPad Software, Inc., La Jolla, CA, USA), n.s. not significant; ^(*)^p < 0.05; ^(**)^p < 0.01; ^(***)^p < 0.001; ^(****)^p < 0.0001.

## Electronic supplementary material

Supplemental Information

Supplementary video 1

Supplementary video 2

Supplementary video 3

Supplementary video 4

Supplementary video 5

Supplementary video 6

Supplementary video 7
