# Supplementary material for: Optical visualisation of thermogenesis in stimulated single-cell brown adipocytes
Source: Sci Rep. 2017 May 3;7:1383. doi: 10.1038/s41598-017-00291-9 (PMC5431191; doi:10.1038/s41598-017-00291-9)
Supplement: Supplementary file 1 — Supplemental Information [file 41598_2017_291_MOESM1_ESM.pdf]

SUPPLEMENTAL INFORMATION

Optical visualisation of thermogenesis in stimulated single-cell brown adipocytes

Rókus Kriszt[1,2,3], Satoshi Arai[4,5], Hideki Itoh[6,7], Michelle H. Lee[2], Anna G. Goralczyk[1,2], Xiu Min Ang[1,2,3], Aaron M. Cypess[8,9], Andrew P. White[10], Farnaz Shamsi[8], Ruidan Xue[8], Jung Yeol Lee[11], Sung-Chan Lee[12,#], Yanyan Hou[4], Tetsuya Kitaguchi[4,5], Thankiah Sudhaharan[13], Shin'ichi Ishiwata[4,5,14], E. Birgitte Lane[7], Young-Tae Chang[11,12], Yu-Hua Tseng[8*], Madoka Suzuki[4,5,15*] and Michael Raghunath[1,2,16,*]

[1]Department of Biomedical Engineering, National University of Singapore, 117583, Singapore

[2]NUS Tissue Engineering Program, Life Science Institute, National University of Singapore, 117510, Singapore

[3]NUS Graduate School for Integrative Sciences and Engineering (NGS), National University of Singapore, 117456, Singapore

[4]WASEDA Bioscience Research Institute in Singapore (WABIOS), 138667, Singapore

[5]Organization for University Research Initiatives, Waseda University, Tokyo, 162-0041, Japan

[6]Department of Pure and Applied Physics, Graduate School of Advanced Science and Engineering, Waseda University, Tokyo 169-8555, Japan

[7]Epithelial Biology Laboratory, Institute of Medical Biology (IMB), Agency for Science, Technology and Research (A*STAR), 138648, Singapore

[8]Section on Integrative Physiology and Metabolism, Joslin Diabetes Center, Harvard Medical School, Boston, MA 02215, USA

[9]Diabetes, Endocrinology, and Obesity Branch, National Institute of Diabetes and Digestive and Kidney Diseases, National Institutes of Health, Bethesda, MA, 20892, USA

[10]Department of Orthopedic Surgery, Beth Israel Deaconess Medical Center, Harvard Medical School, Boston, MA, 02215, USA

[11]Department of Chemistry & MedChem Program of Life Sciences Institute, National University of Singapore, 117543, Singapore

[12]Laboratory of Bioimaging Probe Development, Singapore Bioimaging Consortium (SBIC), Agency for Science, Technology and Research (A*STAR), 138667, Singapore

[13]Institute of Medical Biology, Agency for Science, Technology and Research (A*STAR), 138648, Singapore

[14]Department of Physics, Faculty of Science and Engineering, Waseda University, Tokyo 169-8555, Japan

[15]PRESTO, Japan Science and Technology Agency, 4-1-8 Honcho, Kawaguchi, Saitama, 332-0012, Japan

[16]Department of Biochemistry, Yong Loo Ling School of Medicine, National University of Singapore, 117597, Singapore

***Corresponding authors:**

Michael Raghunath
Email: ragh@zhaw.ch

Madoka Suzuki
Email: suzu_mado@aoni.waseda.jp

Yu-Hua Tseng, Ph.D.
E-mail: yu-hua.tseng@joslin.harvard.edu

[#]Sung-Chan Lee's current affiliation: Aptabio Therapeutics Inc., Yongin City, 446-908, Korea

SUPPLEMENTAL MATERIALS AND METHODS

Chemical synthesis and characterization of ERthermAC

All reactions were performed in oven-dried glassware under a positive pressure of nitrogen (for reaction equation, see Fig. S1A). Unless otherwise noted, starting materials and solvents were purchased from Aldrich and Acros organics and used without further purification. Analytical TLC was carried out on Merck 60 F_{254} silica gel plate (0.25 mm layer thickness) and visualization was done with UV light. Column chromatography was performed on Merck 60 silica gel (230 - 400 mesh).

NMR spectra were recorded on a Bruker Avance 500 MHz NMR spectrometer. Chemical shifts are reported as δ in units of parts per million (ppm) and coupling constants are reported as a *J* value in Hertz (Hz). Mass of ERthermAC compound was determined by LC-MS of Agilent Technologies with an electrospray ionization source. Spectroscopic measurements were performed on a fluorometer and UV/VIS instrument, Synergy 4 of bioteck company and Gemini XS fluorescence plate reader. Relative quantum yield was calculated by comparing the areas under the corrected emission spectrum. The following equation was used to calculate quantum yield:

$$\Phi_x = \Phi_{st}(I_x/I_{st})(A_{st}/A_x)(\eta_x^2/\eta_{st}^2)$$

where Φ_{st} is the reported quantum yield of the standard, I is the integrated emission spectrum, *A* is the absorbance at the excitation wavelength, and *η* is the refractive index of the solvents used. The subscript "x" denotes unknown and "st" denotes standard. Rhodamine B was used as standard.

The relationship between temperature and fluorescence intensity of ERthermAC staining was assessed in WT-1 cells. The cells were fixed with formaldehyde and then placed in a temperature-controlled stage-top incubator attached to an Olympus FV 1000 confocal microscope with a 60× objective (PLAPON60XO, NA 1.42, oil immersion). The temperature of the incubator was gradually changed (0.5°C/min)

and images were taken meanwhile. Temperature of the culture medium was recorded by a thermocouple (6-9248-03, AS ONE, Japan) and a digital thermometer (midi LOGGER GL10-TK, Graphtec, Japan). As the cells were fixed we assumed that the intracellular temperature was equal to the temperature of the medium.

Quantitative PCR

Expression of pan-adipocyte and brown adipose tissue markers were assessed by quantitative real-time PCR (qRT-PCR). For mouse cells, total RNA was extracted from monolayers and subjected to reverse-transcription, followed by 35 cycles of qRT-PCR using Maxima SYBR Green/ROX qPCR Master Mix (K0222, Fermentas, Thermo Fisher Scientific Inc., Rockford, IL, USA) as previously described[1,2]. For human cells, RNA extraction, cDNA synthesis, and qRT-PCR were performed as described before[3]. qRT-PCR assays were run in duplicate and quantified in the ABI Prism 7900 sequence-detection system using SYBR Green (Roche Applied Science, Indianapolis, IN, USA). Relative gene expression levels were determined using the $\Delta\Delta$-Ct method for both species. Results were normalised to ribosomal phosphoprotein P0 (*rplp0,* mouse cells) or to TATA-binding protein (*TBP*, human cells). The primers sequences can be found in Supplementary Table S1 (mouse) and S2 (human).

Measurement of oxygen consumption (OCR) and extracellular acidification rates (ECAR) with Seahorse Extracellular Flux Analyzer

WT-1 cells were seeded into Seahorse 24-well plates at a cell density of 3×10^4 cells/cm^2 and differentiated as described above. Cells were incubated in 500 µl of XF assay medium (Seahorse Bioscience, Chicopee, MA, USA) supplemented with 4.5 g/l D-glucose (BIO-1100, 1st Base, Axil Scientific Pte Ltd, Singapore) and 110 mg/l sodium pyruvate (11360-70, Gibco/Life Technologies) for 20 min at 37°C in the absence of CO_2 before loading into the Seahorse XF^{e}24 Extracellular Flux Analyzer

to measure oxygen consumption rate (OCR) and extracellular acidification rate (ECAR). Cells were stimulated with 10 µM isoproterenol or XF assay medium (vehicle control) for 140 min. To minimise well-to-well variations, raw OCR and ECAR results were normalised to 1 µg double-strand DNA, measured using Quant-iT PicoGreen dsDNA Kit (MP7581, Molecular Probes, Life Technologies).

Seahorse measurements with human brown adipocytes were performed similarly as above described, with the following modifications: cell density was 10.5k cells/cm^2, a Seahorse XF24 Extracellular Flux Analyzer was used and the cells were stimulated with 10 µM forskolin (F6886, Sigma-Aldrich) or DMSO in XF assay medium (vehicle control). To minimise well-to-well variations, raw OCR and ECAR results were normalised to 1 µg protein, measured using Pierce BCA Protein Assay Kit (Thermo Fischer Scientific).

Isothermal microcalorimetry

Calorimetric measurements were performed using the calScreener multi-channel microcalorimeter (SymCel Sverige AB, Kista, Sweden) as recently described[5,6]. Plastic vials provided by the manufacturer, which allow heat measurements in adherent cell cultures (calPlate system), were first coated with 50 µl soluble collagen I (3 mg/ml, IAC-30, KOKEN Cosmo Bio Co. Ltd., Japan) to support cell attachment. WT- cells were seeded at a density of 4 × 10^4/vial, and cultured and differentiated as described above. Immediately prior to calorimetric measurements, medium was replaced with either 300 µl pre-warmed, fresh HG DMEM with GlutaMax, 110 mg/l pyruvate, 10% FBS, 1% P/S, and 10 mM HEPES, or the same medium containing 10 µM isoproterenol. Vials containing the same medium but without cells were used for reference. Samples were placed into titanium vials, and the lids, fitted with O-ring seals, were tightly closed with an adjustable torque screwdriver. Closed vials were positioned in a plastic well-plate format sample holder, which was placed into the

instrument. Samples were first cycled through a three-step thermal equilibration procedure, lasting approximately 1 h, as recommended by the manufacturer. The thermostat was set at 37°C for all experiments. Experiments were repeated twice, with 16 replicates per condition in each run. As a result of technical errors with the reference vials, two samples were omitted from both vehicle and test groups, resulting in 14 replicates per condition. Thermal power data was collected and analysed between 75 and 375 min post-stimulation using CalView software (SymCel Sverige AB, Kista, Sweden).

pH measurements with an ER-specific pHluorin probe

ER-pHluorin was generated by super ecliptic pHluorin[7] inserting between the N-terminal calreticulin ER-targeting signal and the C-terminal KDEL ER-retention signal in pcDNA3 vector, and named pcDNA3-ER-pHluorin. WT-1 cells were transfected with pcDNA3-ER-pHluorin after the 2-day induction step using Lipofectamine 2000. pH-intensity relationship of ER-specific pHluorin was determined by a method originally described[8]: transfected WT-1 cells were perfused with a potassium-rich, pH-clamping buffer (125 mM KCl, 20 mM NaCl, 110 mM Hepes, 10 mM MES (2-(N-morpholino)ethanesulfonic acid), 0.5 mM $CaCl_2$, 0.5 mM $MgCl_2$) containing 5 µM nigericin (an antibiotic drug that is able to equalize the pH across biological membranes) with pH set to 5.5, 7.0 and 8.5. To estimate pH values in ER, a linear was fitted on 7.0 and 8.5 pH data points (intensity = 0.6319*pH - 3.7051). To measure pH fluctuation in ER upon adrenergic stimulation, transfected cells were stimulated with either 10 µM ISO or vehicle and imaged similarly as described in the main text.

Gene	Sequence	Reference
ucp1	Forward: CTTTGCCTCACTCAGGATTGG<br>Reverse: ACTGCCACACCTCCAGTCATT	4
cidea	Forward: TGCTCTTCTGTATCGCCCAGT<br>Reverse: GCCGTGTTAAGGAATCTGCTG	5
pgc1a	Forward: AGCCGTGACCACTGACAACGAG<br>Reverse: GCTGCATGGTTCTGAGTGCTAAG	4
leptin	Forward: CCTCATCAAGACCATTGTCACC<br>Reverse: TCTCCAGGTCATTGGCTATCTG	1
pparg2	Forward: GCATGGTGCCTTCGCTGA<br>Reverse: TGGCATCTCTGTGTCAACCATG	4
fabp4	Forward: ACACCGAGATTTCCTTCAAACTC<br>Reverse: CCATCTAGGGTTATGATGCTCTTC	6
rplp0	Forward: TTTGGGCATCACCACGAAAA<br>Reverse: GGACACCCTCCAGAAAGCGA	1

Supplementary table 1. Primer sequences used for qPCR measurements (mouse). *ucp1*: uncoupling protein 1; *cidea*: cell death-inducing DFFA-like effector A; *pgc1a*: peroxisome proliferator-activated receptor-γ coactivator 1-α; *leptin*: leptin; *pparg2*: peroxisome proliferator-activated receptor-γ 2; *fabp4*: fatty acid binding protein 4; *rplp0*: ribosomal phosphoprotein P0.

Gene	Sequence
UCP1	Forward: ACCGCAGGGAAAGAAACAGC<br>Reverse: TCAGATTGGGAGTAGTCCCT
PGC1A	Forward: AGTGGTGCAGTGACCAATCA<br>Reverse: CTGCTAGCAAGTTTGCCTCA
DIO2	Forward: AGTGCAGAAGGAGGTGACAACAGT<br>Reverse: AAAGTCAAGAAGGTGGCATGTGGC
FAS	Forward: GCATCTGGACCCTCCTACCT<br>Reverse: TCCTCAATTCCAATCCCTTG
FABP4	Forward: ACTGGGCCAGGAATTTGACGAAGT<br>Reverse: TCTCGTGGAAGTGACGCCTTTCAT
PPARG2	Forward: ACTCTGGGAGATTCTCCTATT<br>Reverse: CTCCATAGTGAAATCCAGAAG
TBP	Forward: CACGAACCACGGCACTGATT<br>Reverse: TTTTCTTGCTGCCAGTCTGGAC

Supplementary table 2. Primer sequences used for qPCR measurements (human). *UCP1*: uncoupling protein 1; *PGC1A*: peroxisome proliferator-activated receptor-γ coactivator 1-α; *DIO2*: Type II iodothyronine deiodinase; *PPARG2*: peroxisome proliferator-activated receptor-γ 2; *FAS*: fatty acid synthase; *FABP4*: fatty acid binding protein 4; *TBP*: TATA-binding protein.

SUPPLEMENTAL RESULTS

Chemical characterization

Quantum Yield (Φ): 0.20 (Solvent: DMSO, Reference Dye: Rhodamine B, Excitation wavelength (λ_{ex}): 510nm) (Fig. S1B)

Extinction coefficient (ε): 53940 $M^{-1}cm^{-1}$ (Solvent: DMSO, Wavelength (λ): 565 nm) (Fig. S1B).

^{1}H NMR ($(CD_3)_2SO$, 500 MHz): δ 10.23 (s, 1H), 7.88 (d, *J* = 16.4 Hz, 1H), 7.80 (d, *J* = 8.4 Hz, 2H), 7.76 (d, *J* = 5.8 Hz, 1H), 7.42 (d, *J* = 8.6 Hz, 3H), 7.25 (d, *J* = 10.3 Hz, 1H), 7.14(s, 1H), 7.02 (d, *J* = 8.9 Hz, 1H), 6.47 (dd, *J* = 15.6, 3.4 Hz, 2H), 5.75 (s, 1H), 2.12 (s, 3H), 1.64 (s, 3H).

^{13}C NMR ($(CD_3)_2SO$, 125 MHz): δ 15.6, 24.6, 55.3, 116.9, 118.1, 119.0, 119.7, 120.3, 121.8, 124.0, 124.3, 126.6, 128.1, 130.1, 134.8, 135.0, 136.1, 138.5, 141.1, 141.2, 142.2, 146.2, 156.7, 158.4, 169.2 (Fig. S2).

ESI-MS *m/z* (M+H) calc'd: 542.2, found 542.0 (Fig. S3).

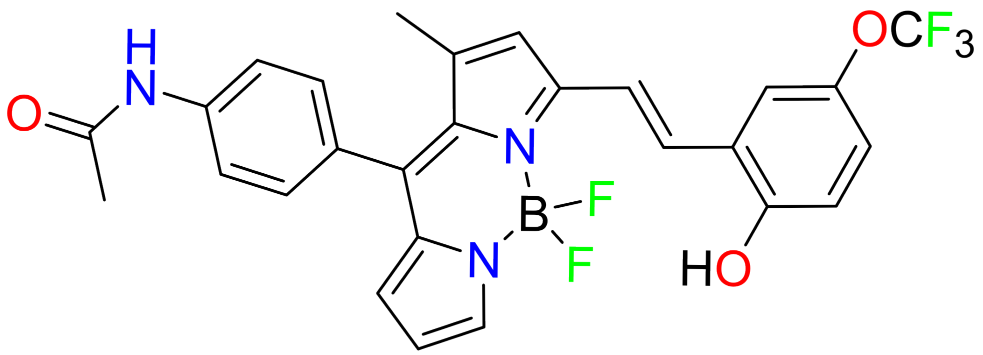

Chemical formula: $C_{27}H_{21}BF_5N_3O_3$
Exact mass: 541.16
Molecular weight: 541.28

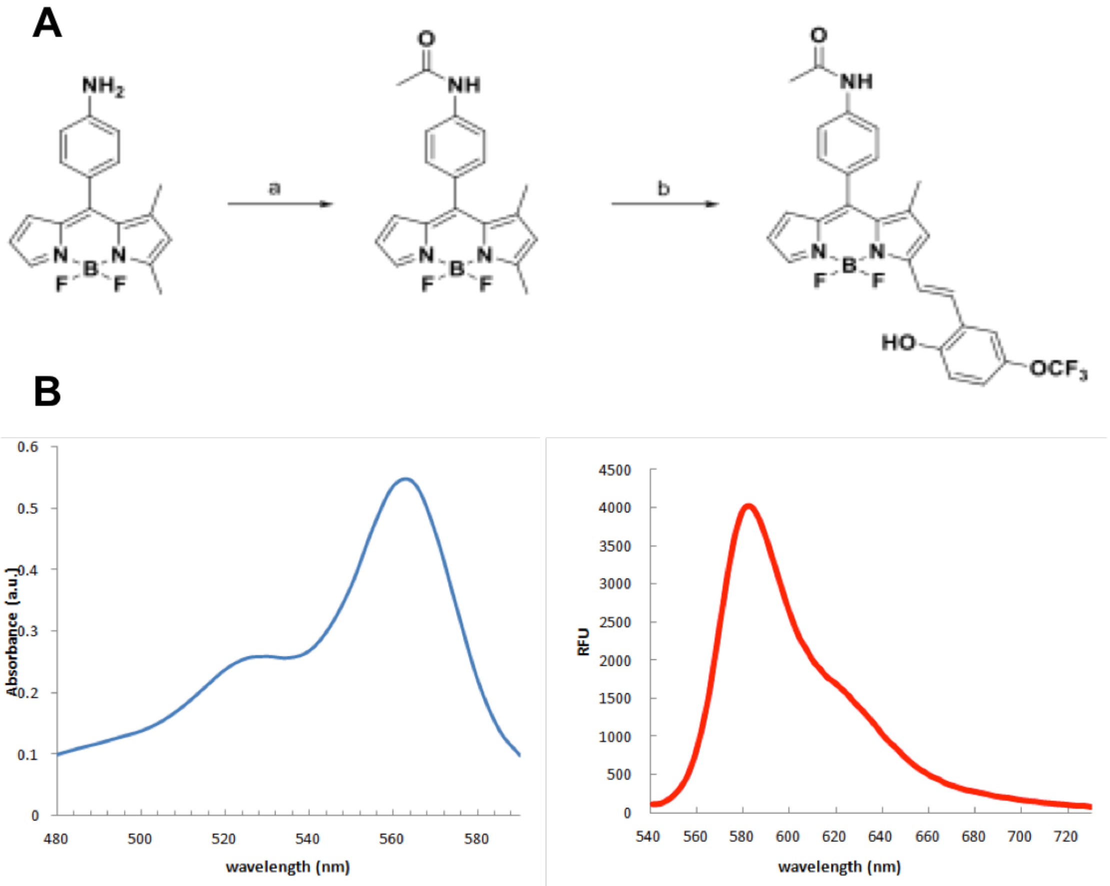

Supplementary figure 1. Synthetic scheme (A) and absorption and emission spectra (B) of ERthermAC. **(A)** a) acetyl chloride, Et_3N, 30 min in DCM. b) 3-threefluoromethoxy-6-hydroxy-benzaldehyde, pyrrolidine, AcOH, $95^{o}C$, 10 min in acetonitrile. **(B)** Spectra were measured at the concentration of 10 μM in DMSO, excitation wavelength (λ_{ex}) = 510nm.

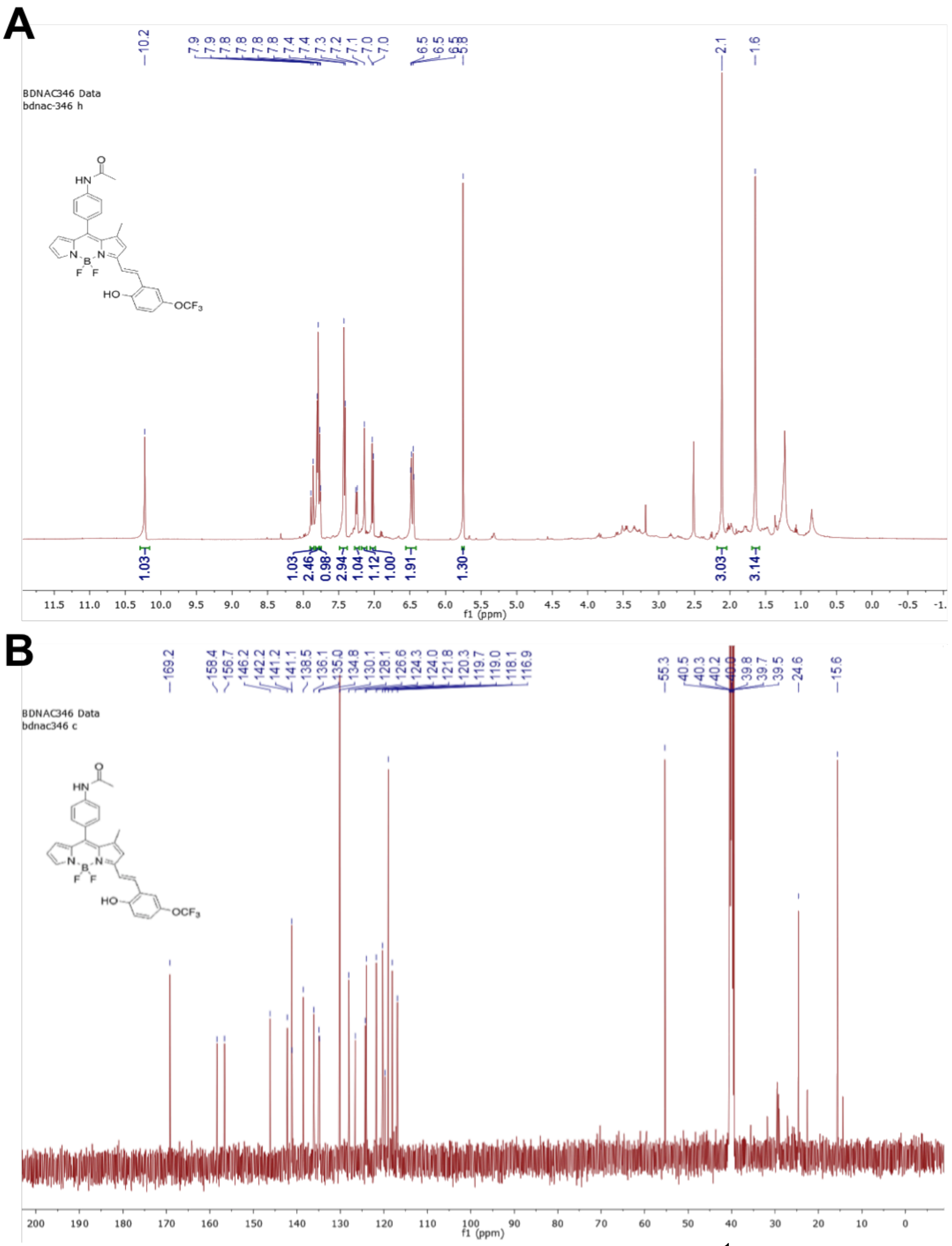

Supplementary figure 2. NMR spectra of ERthermAC. (A) ^{1}H NMR (500 MHz, $(CD_3)_2SO$) spectrum. **(B)** ^{13}C NMR (125 MHz, $(CD_3)_2SO$) spectrum.

A

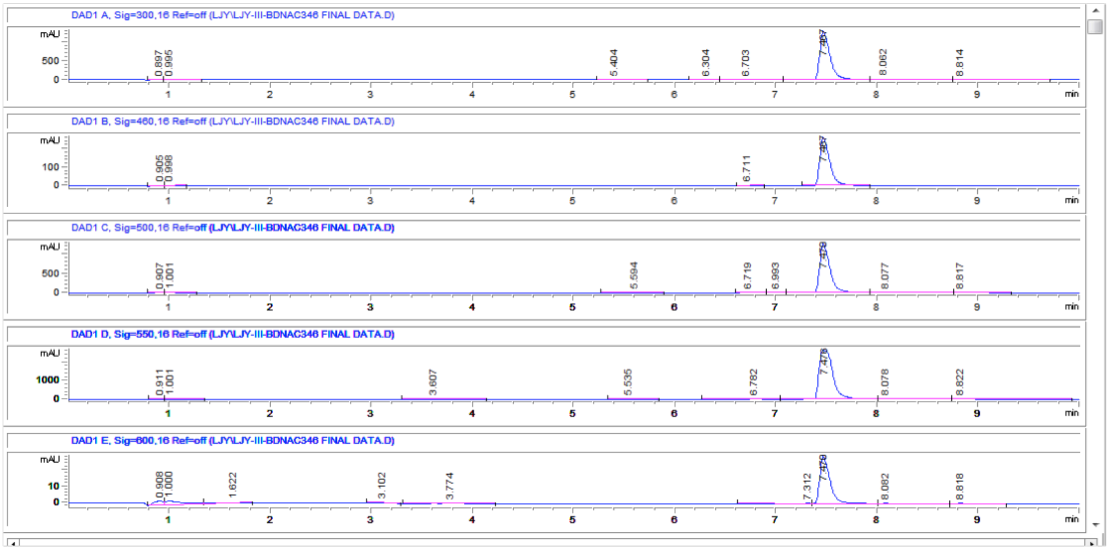

B

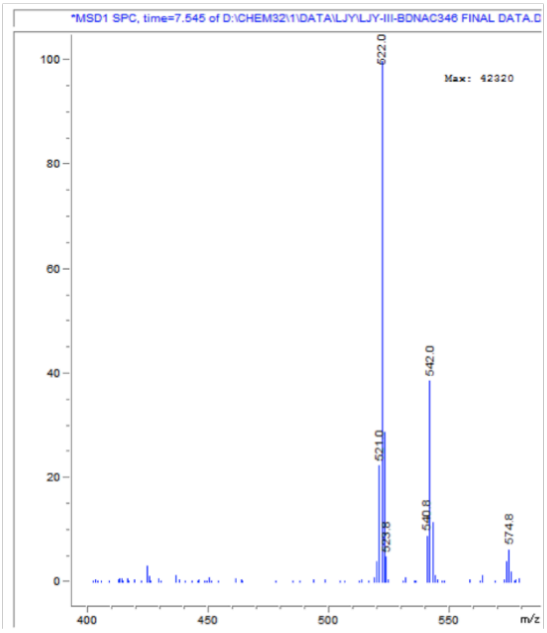

Supplementary figure 3. HPLC chromatogram (A) and mass spectrum (B) of ERthermAC.

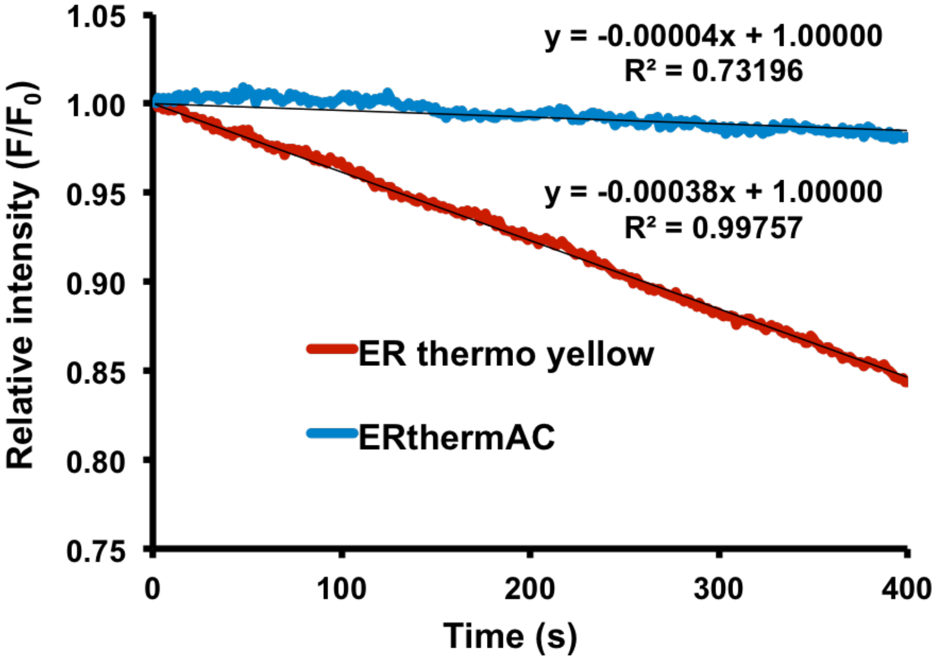

Supplementary figure 4. Photostability of ERthermAC and ER thermo yellow. Cells were continuously irradiated by higher-power (17.14 μW), 543 nm laser, and images were taken at every 1.4 s sequentially. In the given time-frame, ER thermo yellow bleached approximately ten times faster than ERthermAC.

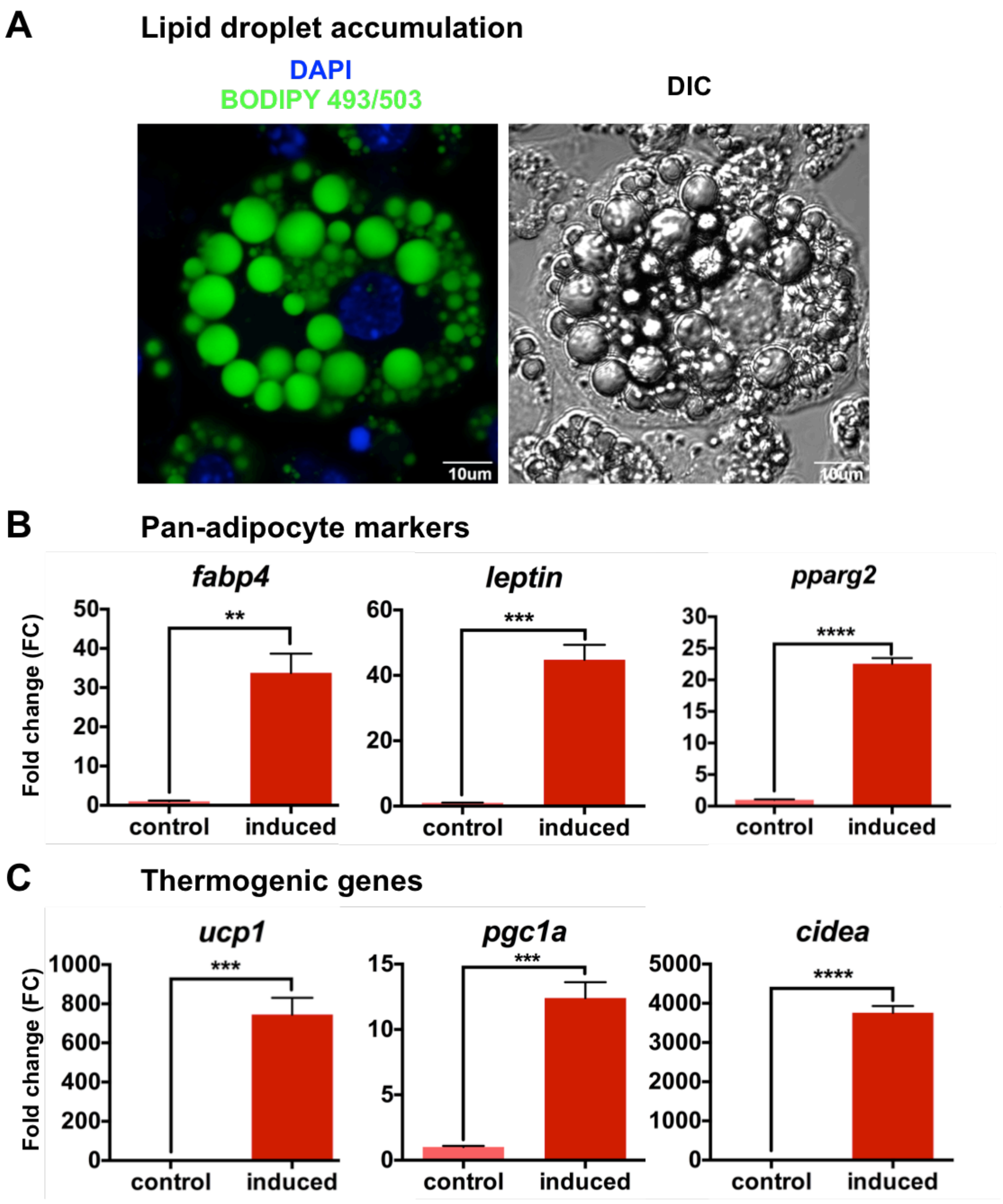

Supplementary figure 5. Characterisation of WT-1 cells. (A) After induction, dense lipid droplet formation occurs in WT-1 cells (green: BODIPY 493/503; blue: DAPI) suggesting successful adipogenic differentiation. Scale bar: 20 μm. **(B)** mRNA levels of pan-adipocyte markers *leptin*, *fabp4*, and *pparg2* are significantly increased in induced WT-1 Cells, compared with respective controls. Results are expressed as mean ± SEM (n = 3 for all groups). **(C)** Gene expression of thermogenic gene *ucp1*, *pgc1a,* and *cidea* is elevated in induced WT-1 cells, compared with undifferentiated precursor cells. Results are expressed as mean ± SEM (n = 3 cultures for all groups), (*) $p < 0.05$; (**) $p < 0.01$; (***) $p < 0.001$; (****) $p < 0.0001$.

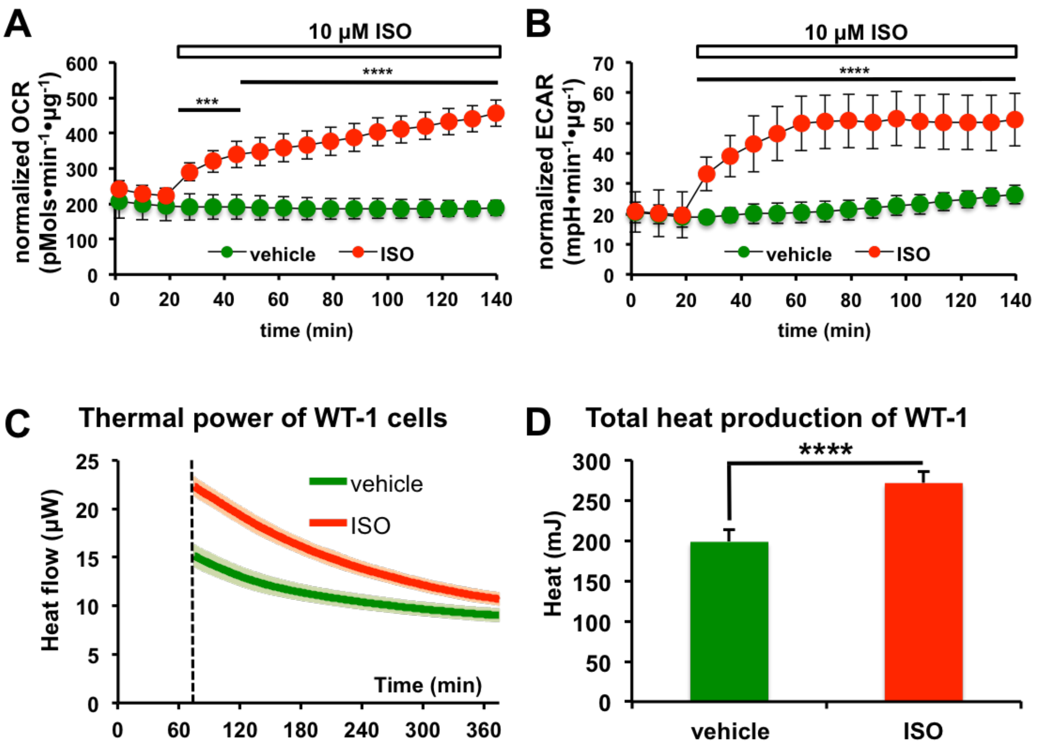

Supplementary figure 6. Isoproterenol stimulates oxygen consumption, extracellular acidification and heat production in WT-1 cells. (A) Oxygen consumption rate (OCR) is drastically increased in WT-1 cells after adrenergic stimulus (ISO). Results are expressed as mean ± SD, normalised to 1 µg double-strand DNA. WT-1 vehicle: n = 5 cultures; WT-1 ISO: n = 3 cultures, (***) $p < 0.001$; (****) $p < 0.0001$. **(B)** ISO enhances glycolysis in WT-1 cells. Results are expressed as mean ± SD, normalised to 1 µg double-strand DNA. WT-1 vehicle: n = 5 cultures; WT-1 ISO: n = 3 cultures, (****) $p < 0.0001$. **(C)** Average thermal power of WT-1 cells after vehicle (green) or ISO (red) stimulation. Results are expressed as mean ± SD. WT-1 vehicle: n = 14; WT-1 ISO: n = 14. **(D)** Average total heat production of WT-1 cells between 75 min and 375 min post stimulation. Results are expressed as mean ± SD. WT-1 vehicle: n = 14 cultures; WT-1 ISO: n = 14 cultures, (****) $p < 0.0001$.

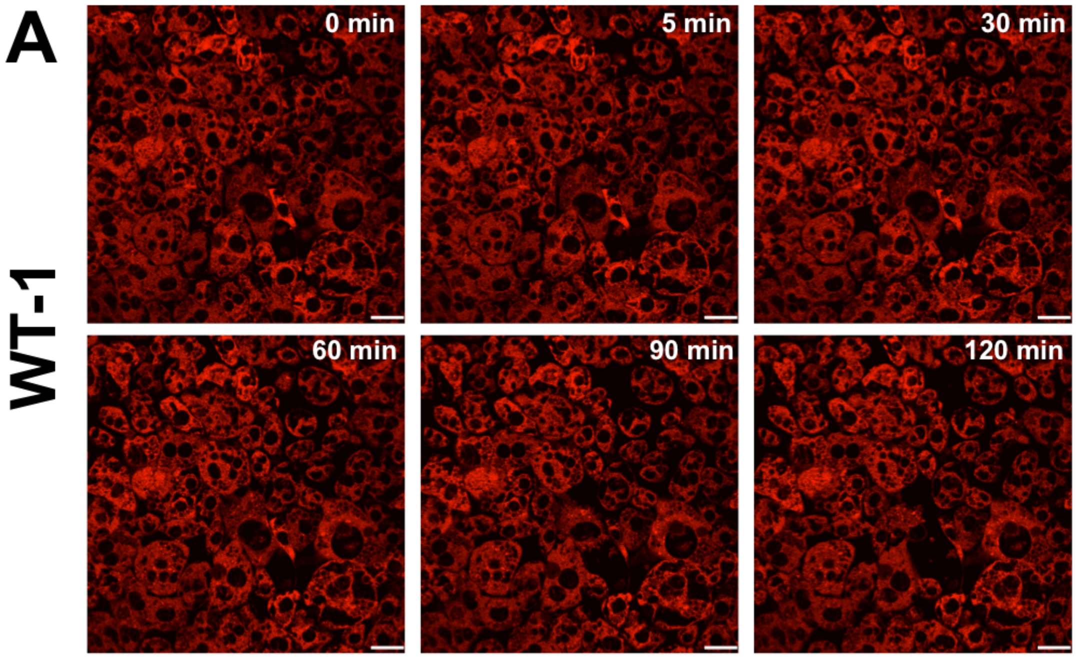

Supplementary figure 7. Vehicle stimulation does not affect the intensity of ERthermAC in WT-1 cells. Scale bar: 20 μm.

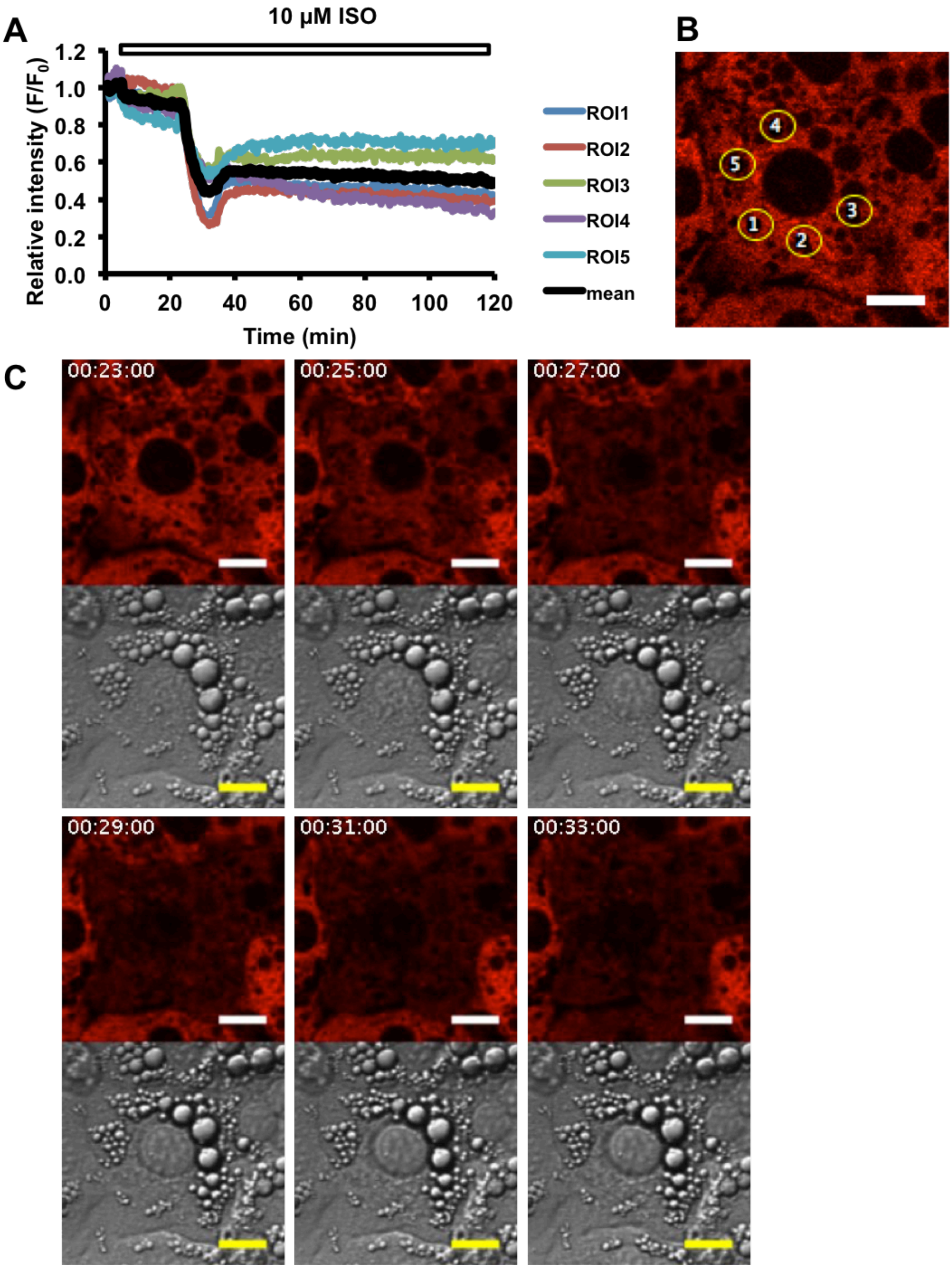

Supplementary figure 8. ERthermAC intensity change of a representative ISO-stimulated WT-1 cell. (A) Fluorescence of ERthermAC at different regions of interest (ROIs) **(B)** drastically decreases in ISO-stimulated WT-1 cells. Drop of intensity occurs after a lag phase and this process lasts for approximately 8 minutes. Then, the intensity remains low until the end of the experiment suggesting sustained heat production. Thick black curves correspond to average relative intensity of different ROIs. **(C)** Differential interference contrast (DIC) images show slight morphological change meanwhile ERthermAC intensity is changing. Scale bar: 10 µm. For time-lapse video, see Supplementary video 2.

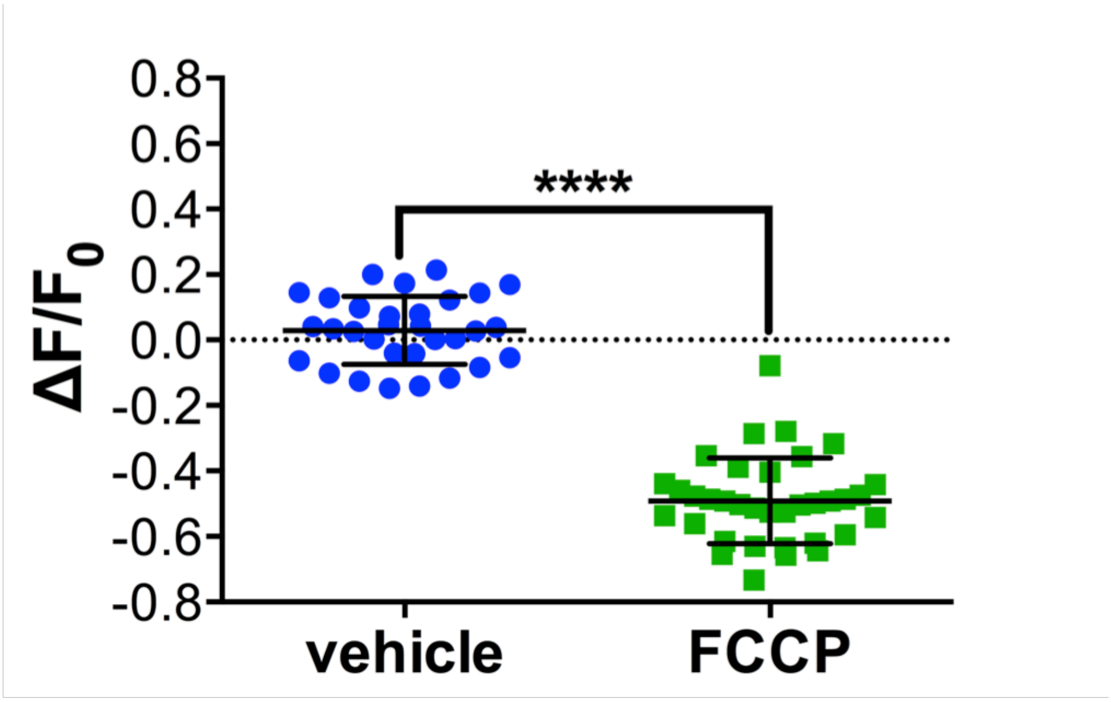

Supplementary figure 9. Scatter plot of ERthermAC relative intensity change after FCCP stimulation in WT-1 cells. ERthermAC intensity significantly decreases upon FCCP stimulation ($p<0.0001$). Vehicle: n=31 cells, N=2 cultures; FCCP: n=35 cells, N=2 cultures.

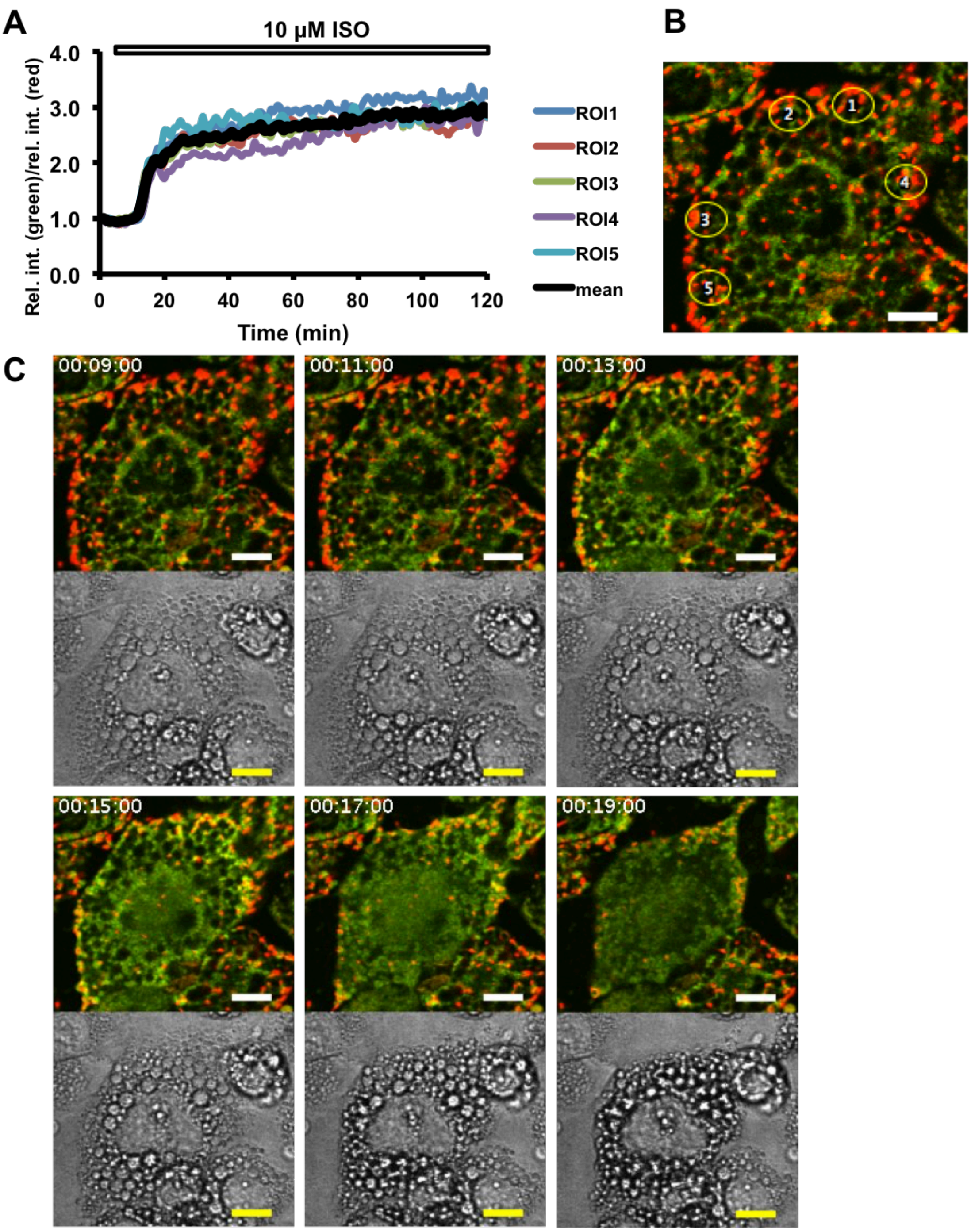

Supplementary figure 10. JC-1 color change of a representative ISO-stimulated WT-1 cell. (A) The change in relative intensity ratio of green (JC-1 monomers) and red (JC-1 aggregates) indicates fast mitochondrial depolarization in ISO-stimulated WT-1 cells, at different regions of interest (ROIs) **(B)**. Red-to-green transition occurs after a lag phase and lasts for approximately 10 minutes. Then, the ratio of green and red fluorescence remains higher until the end of the experiment suggesting elevated mitochondrial membrane potential. Thick black curves correspond to average relative intensity ratio of green (JC-1 monomers) and red (JC-1 aggregates) signals of different ROIs. **(C)** Differential interference contrast (DIC) images show slight morphological change meanwhile the color of JC-1 is changing. Scale bar: 10 μm. For time-lapse video, see Supplementary video 5.

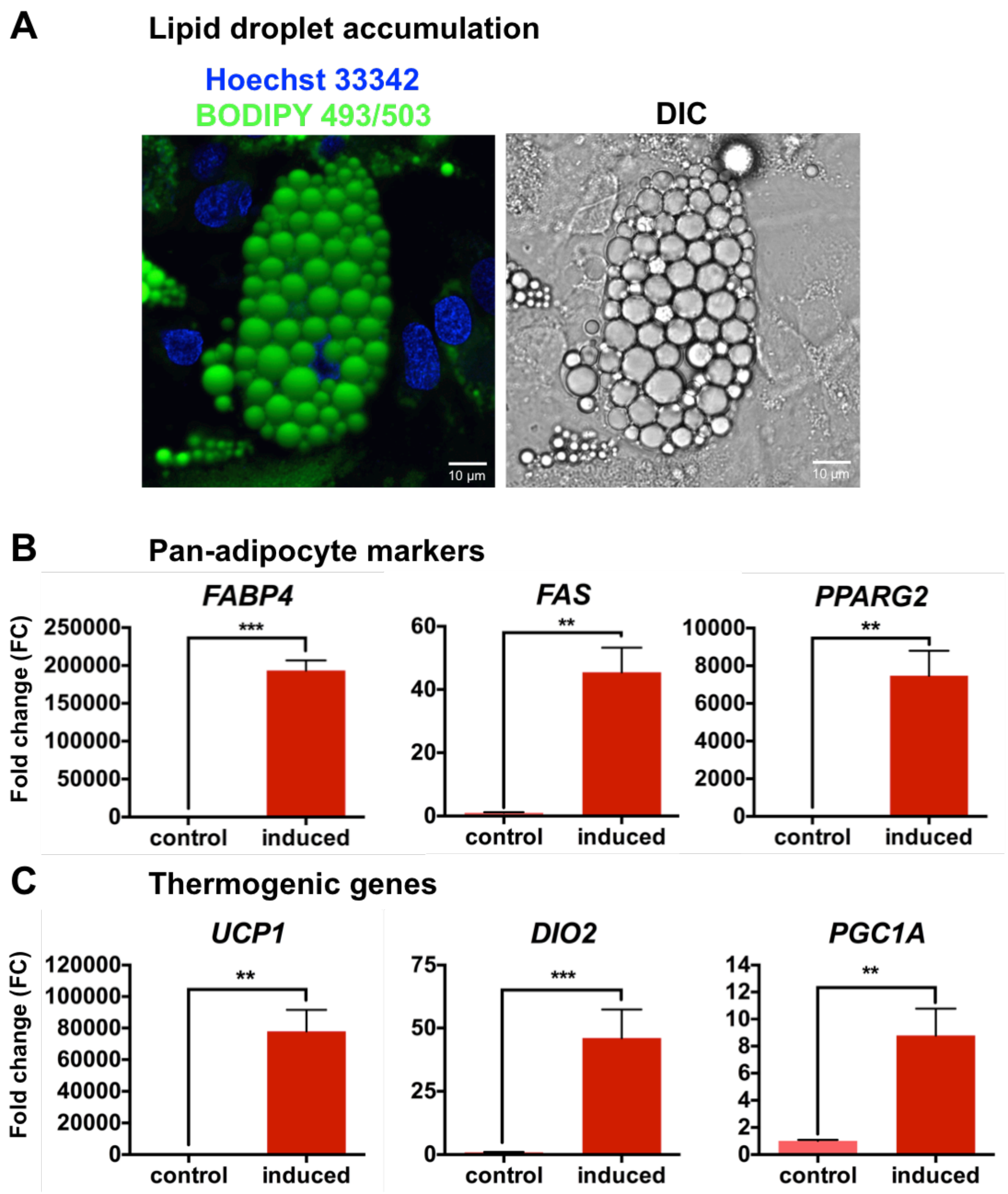

Supplementary figure 11. Characterisation of human brown adipocytes. (A) After induction, dense lipid droplet formation occurs in brown adipocytes (green: BODIPY 493/503; blue: Hoechst 33342) suggesting successful adipogenic differentiation. Scale bar: 10 µm. **(B)** mRNA levels of pan-adipocyte markers *FAS*, *FABP4* and *PPARG2* are significantly increased in induced brown fat cells, compared with undifferentiated controls. Results are expressed as mean ± SEM (n = 4 for all groups). **(C)** Gene expression of thermogenic gene *UCP1, DIO2* and *PGC1A* were elevated in induced brown adipocytes, compared to undifferentiated controls. Results are expressed as mean ± SEM (n = 4 cultures for all groups), (**) $p < 0.01$; (***) $p < 0.001$.

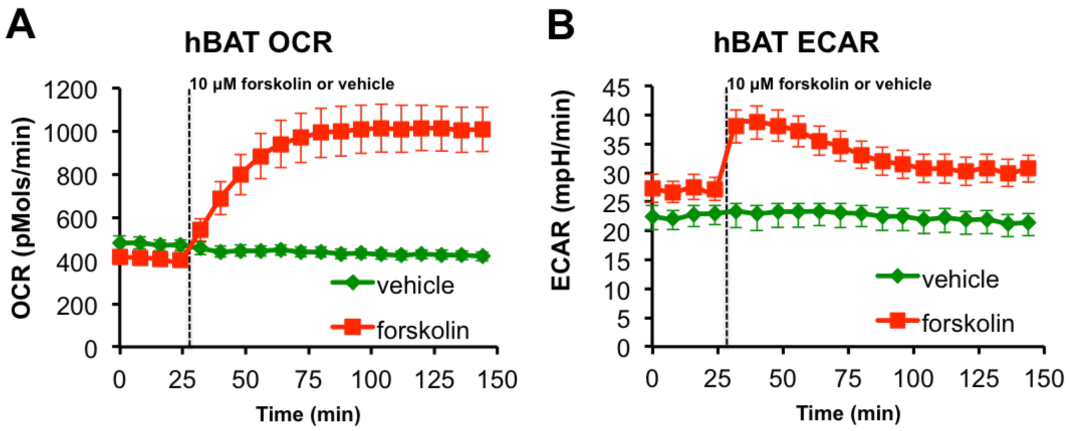

Supplementary figure 12. Forskolin stimulates oxygen consumption and extracellular acidification in human brown adipocytes. (A) Oxygen consumption rate (OCR) increases drastically in human BAs in response to forskolin. **(D)** Forskolin enhances extracellular acidification rate (ECAR) in human BAs. Results are expressed as mean ± SEM. n = 10 for both groups.

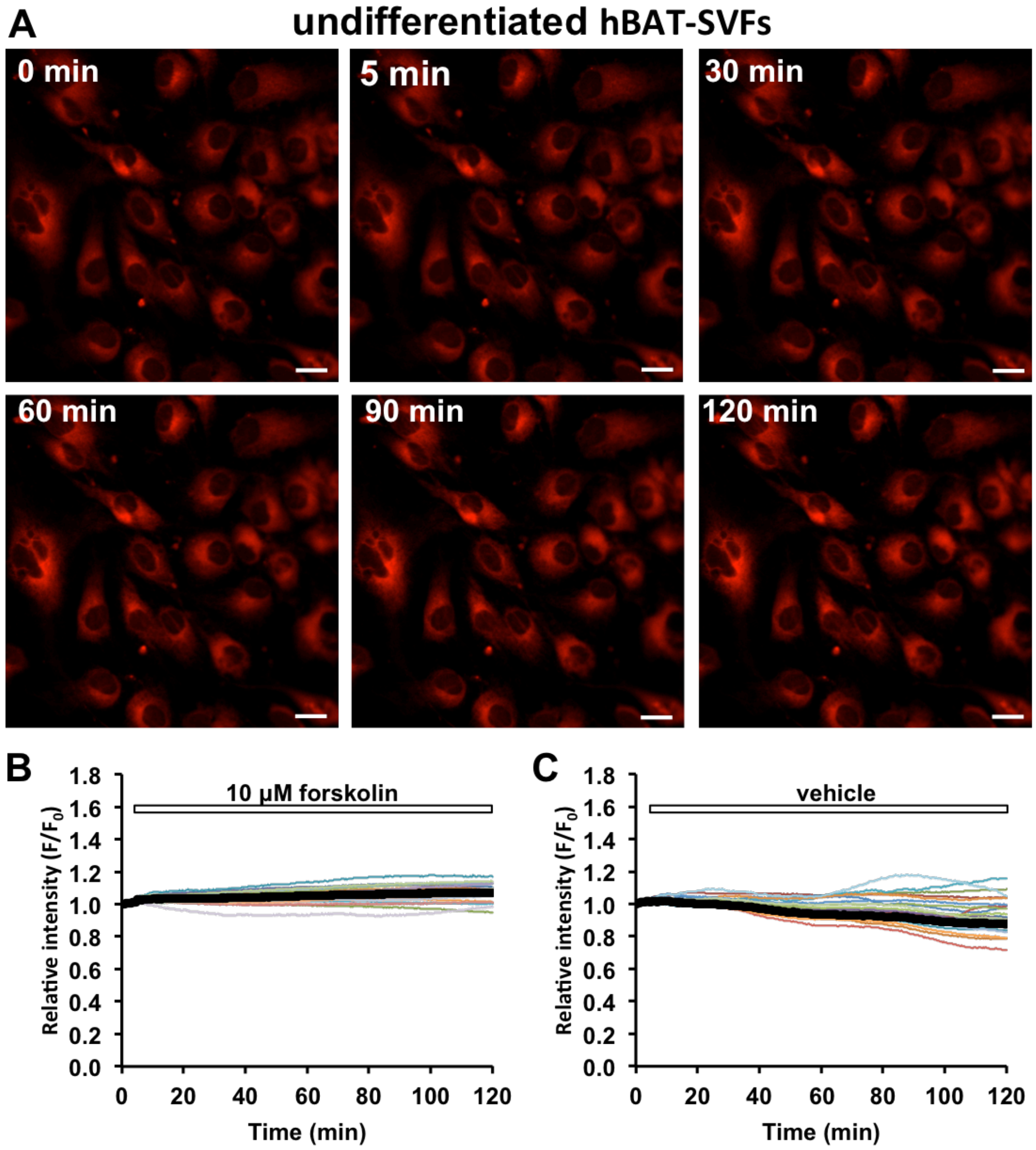

Supplementary figure 13. Undifferentiated human BAT stromal vascular fraction cells do not respond to forskolin stimulation. (A) In undifferentiated human brown preadipocytes, ERthermAC intensity does not change upon forskolin stimulation. Quantitative analysis of ERthermAC fluorescence intensity after forskolin **(B)** and vehicle **(C)** stimulation: individual cells do not exhibit a rapid decline in relative intensity after forskolin stimulation. Thick black curves correspond to the mean relative intensity in each group. Scale bar: 20 µm.

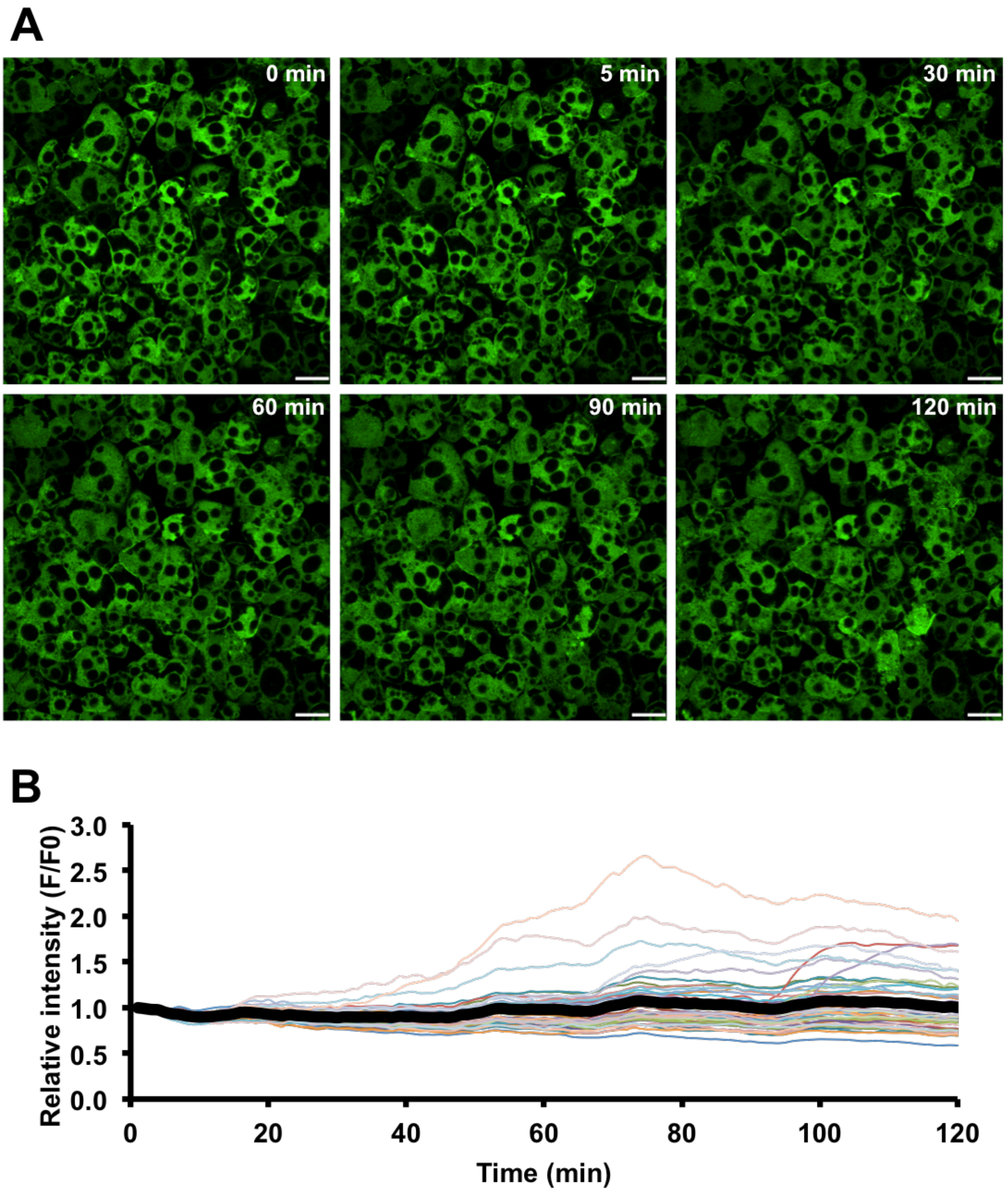

Supplementary figure 14. ISO-stimulated WT-1 cells stained by ER-Tracker Green. (A) ER-Tracker Green is unable to visualize heat production in WT-1 cells upon adrenergic stimulation. **(B)** Thick black curve corresponds to average relative intensity of ER Tracker Green. Scale bar: 20 μm.

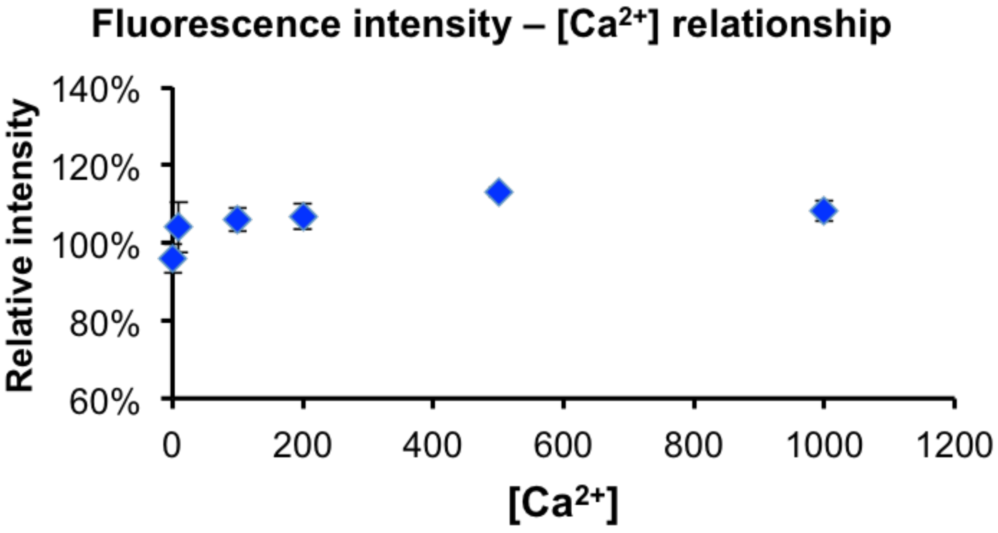

Supplementary figure 15. Effect of Ca^{2+} concentration on ERthermAC intensity in cuvette. The [Ca^{2+}] – fluorescence intensity relationship was determined in HEPES buffer (20 mM, pH=7.5) containing $CaCl_2$ (concentration: 1 µM; 10 µM; 100 µM; 200 µM; 500 µM; 1000 µM) 50 µM BSA and 25 µM ERthermAC (DMSO 0.1%) by spectrophotometry.

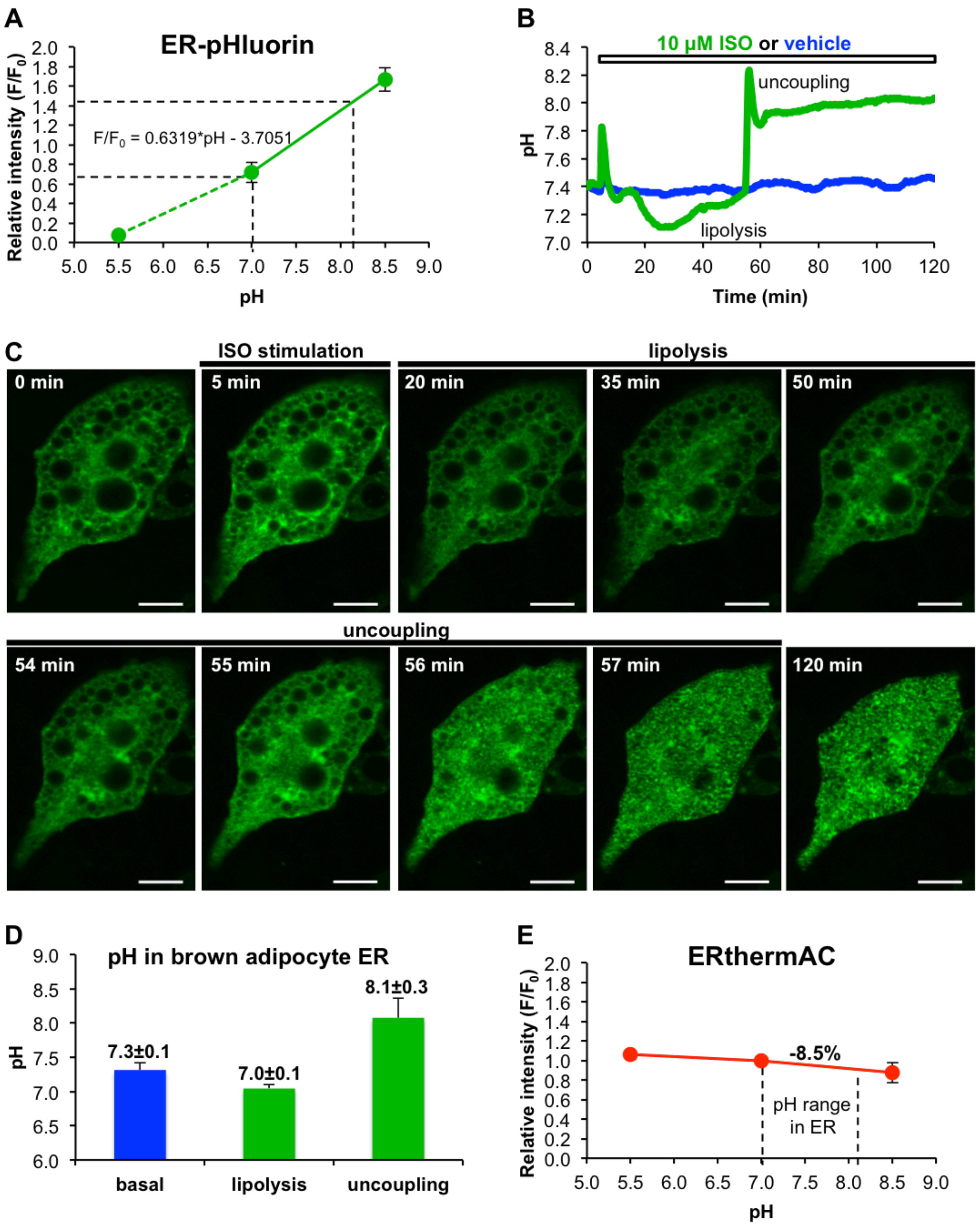

Supplementary figure 16. Changing pH in stimulated brown adipocytes has no major impact on ERthermAC intensity. **(A)** Calibration curve of ER-pHluorin (pH - fluorescence intensity relationship). **(B)** A representative ISO-stimulated WT-1 cell shows slight acidification upon ISO-injection that corresponds to lipolysis. 49 minutes after stimulation, the pH in the ER increased in a prompt manner, that change corresponds to the onset of uncoupled respiration. **(C)** Images of the cell corresponds to the green graph in figure section B. Scale bar: 10 µm. For time-lapse video, see Supplementary video 9. **(D)** Average pH in ER in unstimulated cells (blue, n=6 cells, N=3 cultures) and in stimulated cells (green, n=5 cells; N=4 cultures). Bars of lipolysis and uncoupling represent the average of lowest and highest pH values, respectively. **(E)** Calibration curve of ERthermAC indicates only a moderate change (-8.5%) of intensity in the physiological pH range (7.0-8.1) of the ER in stimulated brown adipocytes.

REFERENCES

1. Lee, M.H. et al. ECM microenvironment unlocks brown adipogenic potential of adult human bone marrow-derived MSCs. *Sci. Rep.* **6,** 21173 (2016).
2. Ang, X.M. et al. Macromolecular crowding amplifies adipogenesis of human bone marrow-derived mesenchymal stem cells by enhancing the pro-adipogenic microenvironment. *Tissue Eng. Part A* **20,** 966-981 (2014).
3. Cypess, A.M. et al. Anatomical localization, gene expression profiling and functional characterization of adult human neck brown fat. *Nat. Med.* **19**, 635–639 (2013).
4. Tseng, Y.H. et al. New role of bone morphogenetic protein 7 in brown adipogenesis and energy expenditure. *Nature.* **454,** 1000-1004 (2008).
5. Braissant, O. et al. Isothermal microcalorimetry accurately detects bacteria, tumorous microtissues and parasitic worms in a label-free well-plate assay. *Biotechnol. J.* **10,** 460-468 (2015).
6. Flores, D., Panic, G., Braissant, O. & Keiser J. A novel isothermal microcalorimetry tool to assess drug effects on Ancylostoma ceylanicum and Necator americanus. *Appl. Microbiol. Biotechnol.* (2015)
7. Sankaranarayanan, S., De Angelis, D., Rothman, J.E. & Ryan, T.A. The use of pHluorins for optical measurements of presynaptic activity. *Biophys. J.* **79,** 2199-2208 (2000).
8. Kim, J.H., Lingwood, C.A., Williams, D.B., Furuya, W., Manolson, M.F. & Grinstein, S. Dynamic measurement of the pH of the Golgi complex in living cells using retrograde transport of the verotoxin receptor. *J. Cell Biol.* **134,** 1387-1399 (1996).
9. Wu, J. et al. Beige adipocytes are a distinct type of thermogenic fat cell in mouse and human. *Cell* **150,** 366-376 (2012).
10. Seale, P. et al. PRDM16 controls a brown/skeletal muscle switch. *Nature* **454,** 947-948 (2008).

11. Rajakumari, S. et al. EBF2 determines and maintains brown adipocyte identity. *Cell Metab.* **17,** 562-574 (2013).

SUPPLEMENTARY VIDEOS

Supplementary video 1. ERthermAC shows robust temperature change after isoproterenol stimulation in WT-1 cells.

Supplementary video 2. ERthermAC intensity and concurrent morphological changes of a representative ISO-stimulated WT-1 cell.

Supplementary video 3. Isoproterenol stimulation induces mitochondrial depolarization in WT-1 cells.

Supplementary video 4. JC-1 color and concurrent morphological changes of a representative ISO-stimulated WT-1 cell.

Supplementary video 5. Forskolin stimulation reduces the intensity of ERthermAC in human brown adipocytes suggesting increased thermogenic activity.

Supplementary video 6. Forskolin stimulation induces mitochondrial depolarization in human brown adipocytes.

Supplementary video 7. Endoplasmic reticulum pH change and concurrent morphological changes of a representative ISO-stimulated WT-1 cell.
